# Total synthesis and biological evaluation of Koshidacin B, TAN-1746, and Ac-TAN-1746

**DOI:** 10.1186/s13065-025-01581-4

**Published:** 2025-07-16

**Authors:** Xiong-En Long, Hailiang Xing, Yixin He, Xuanli Meng, Yanling Ma, Chang Liu, Xi Cao, Huiru Nan, Min-Jing Cheng, Jia-Lei Yan, Junyang Liu

**Affiliations:** 1 School of Pharmacy and Food Engineering, Wuyi University, Jiangmen, 529020 China; 2 Center for Bioactive Natural Molecules and Innovative Drugs, Guangdong Province Key Laboratory of Pharmacodynamic Constituents of TCM and New Drugs Research, College of Pharmacy, Jinan University, Guangzhou, 510632 China

**Keywords:** Total synthesis, Chlamydocin analog, Koshidacin B, TAN-1746, Cross metathesis

## Abstract

Analogues of chlamydocin derivatives consistently demonstrate multifaceted bioactivities, particularly through HDAC inhibition mechanisms, oncolytic efficacy, and neuronal preservation capacities. These activities primarily originate from their unique cyclic tetrapeptide scaffold and functional groups within the side chains that enable specific interactions with biomacromolecules. We herein report the total syntheses of three naturally occurring chlamydocin analogues—koshidacin B, TAN-1746, and Ac-TAN-1746. The synthetic strategy employed a modular approach that involved modifying a common intermediate via late-stage olefin cross-metathesis to install the requisite side-chain fragments. This versatile approach enabled an efficient and divergent total synthesis of the target compounds, completing the syntheses in a longest linear sequence of 9–10 steps. Remarkably, TAN-1746 and Ac-TAN-1746 demonstrated significant anti-osteosarcoma activity that exceeded the potency of clinically employed cisplatin—an observation not previously documented. These results strongly validate the utility of the chlamydocin scaffold as a platform for the development of potent inhibitors with promising therapeutic potential.

**Graphical Abstract:**

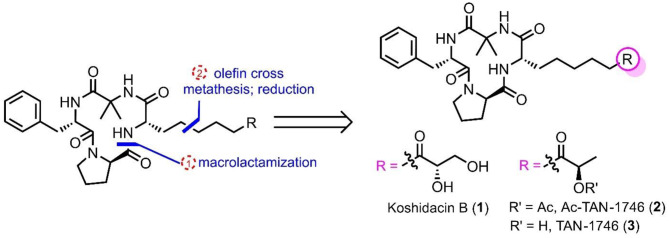

**Supplementary Information:**

The online version contains supplementary material available at 10.1186/s13065-025-01581-4.

## Introduction

Cyclic tetrapeptides (CTPs) represent a captivating class of natural products renowned for their significant biological activities [1]. This structural class is characterized by a strained 12-membered macrocyclic ring, which not only confers resistance to proteases but also enhances the binding affinity towards receptor targets [2]. Aoe-containing CTP family is defined by the presence of the unique Aoe [(2 S,9 S)-2-amino-8-hydroxy-9,10-epoxydecanoic acid] lipophilic amino acid, which features an epoxyketone moiety. The well-known members of this Aoe-containing CTP family include chlamydocin [3], HC-toxin [4], WF-3161 [5], Cyl-1 [6], Cyl-2 [7], and trapoxins [8] etc. These cyclic peptides primarily target histone deacetylases (HDAC), a crucial nuclear enzyme that regulates gene expression through the modulation of acetylation and deacetylation of histone lysines [9, 10]. Notably, chlamydocin was the first member of the Aoe-containing CTP family to be isolated, originally from the fungus *Diheterospora chlamydosporia* [3] with a potent HDAC inhibitory activity (IC_50_ = 1.3 nM) in vitro.

**Fig. 1:**
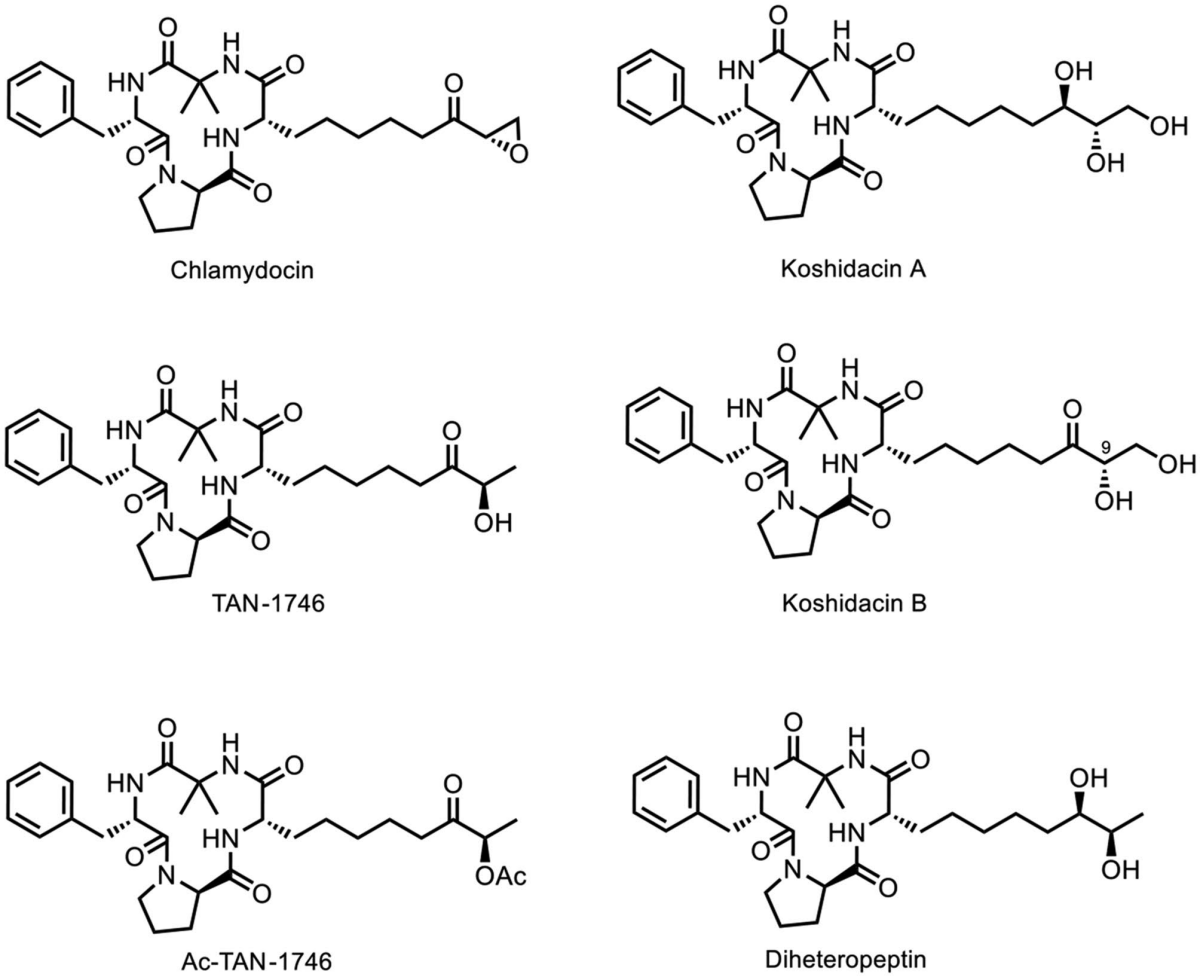
Structures of chlamydocin and its natural analogues

To date, several naturally occurring chlamydocin analogs have been isolated, differing only in the oxidation states of their side chains, as depicted in Fig. 1. TAN-1746, initially isolated by Gupta in 1994 from *Verticillium coccosporum* [11], exhibits potent antitumor activity as an effective HDAC inhibitor [12]. This compound was later isolated by Nakajima and colleagues in 2001 from the fungus *Peniophora sp*., along with Ac-TAN-1746 [13]. Activity studies have shown that TAN-1746 and Ac-TAN-1746 also exhibit activity in suppression of shoot elongation in rice seedlings while preventing chlorotic lesions and turgor loss. In 2022, Koshidacins A and B were isolated by Iwatsuki and co-workers from the Okinawan fungus *Pochonia boninensis* FKR-0564. They display a moderate in vitro antiplasmodial activity against *Plasmodium falciparum* strains [14]. Particularly, Koshidacin B suppressed 41% of malaria parasites in vivo, showing its potential for further antimalarial drug development. The total synthesis of chlamydocin [15–20] and its analogs has been extensively reported [21–24]. These efforts commonly involve the synthesis of the Aeo segment first, followed by macrocyclization to achieve the total synthesis of chlamydocin. Remarkably, in 2019, Kananovich successfully realized a diverse synthesis of chlamydocin and its analogs by employing a late-stage cyclopropane ring cleavage strategy [20], making the synthesis more variable and efficient. The remarkable biological activity and unique structural features of chlamydocin and its natural product analogs have captivated our interest. Therefore, we aim to explore divergent access to a series of chlamydocin structural analogs. To this end, we assumed to synthesize a common cyclic peptide intermediate, utilizing intermolecular olefin cross-metathesis reactions, to converge the synthesis of three bioactive natural occurring chlamydocin analogs, e.g. TAN-1746, Ac-TAN-1746, Koshidacin B, providing a novel pathway for their diverse synthesis. It is worth noting that during the preparation and submission of this manuscript, the Chattopadhyay group, as well as the groups of Chen, Wang, and Sun, independently reported elegant strategies for the synthesis of 9-*epi*-koshidacin B [25] and koshidacins A and B [26], respectively, via late-stage olefin cross-metathesis. These excellent contributions, alongside our work, collectively underscore the efficiency of this synthetic strategy in constructing such complex natural products. Our study was conceived and completed entirely independently, and its novelty is strongly supported by a patent application that was submitted before the publication of these related works [27]. In contrast to the aforementioned studies, which focus specifically on the synthesis of koshidacin derivatives, our research focuses on the development of a general and versatile synthetic platform. Utilizing a common cyclic peptide intermediate, we efficiently accomplished the total synthesis of three structurally related yet biologically distinct natural products—koshidacin B (**1**), Ac-TAN-1746 (**2**), and TAN-1746 (**3**). More importantly, we have, for the first time, identified potent anti-osteosarcoma activity for Ac-TAN-1746 (**2**) andTAN-1746 (**3**), with efficacy markedly surpassing that of the clinically used chemotherapeutic agent cisplatin. In addition, our synthetic route to koshidacin B, featuring a longest linear sequence of only 9 steps, offers a more concise and efficient alternative to previously reported methods (11–12 steps). Overall, our study not only provides an efficient, divergent synthetic route to important natural product analogues but also reveals new therapeutic potential in the field of anticancer drug discovery, thereby significantly expanding the scope of research on chlamydocin-type molecules.

## Results and discussion

**Fig. 2:**
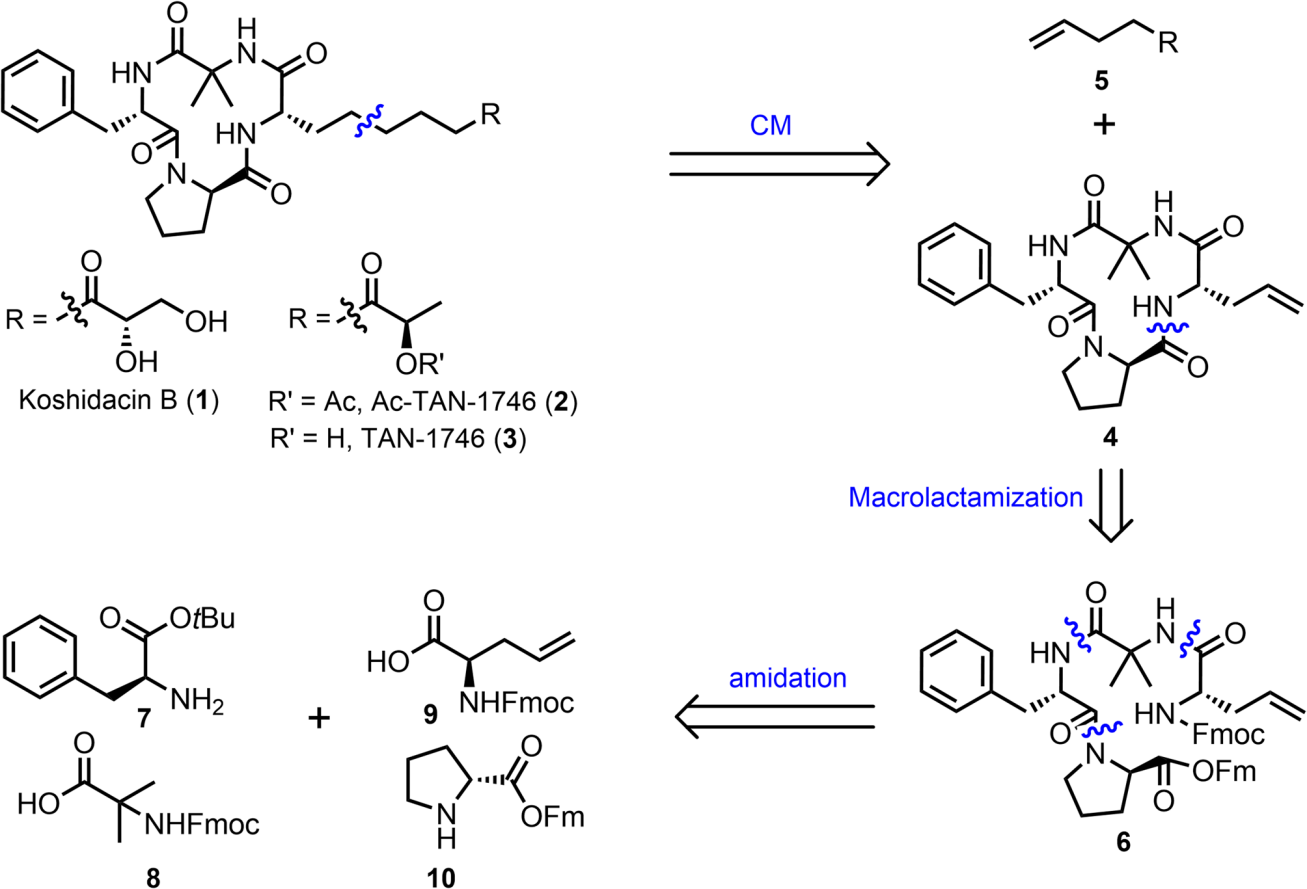
Retrosynthetic analysis chlamydocin and its natural occurring analogs

Our retrosynthetic analysis is outlined in Fig. 2. We planned to employ olefin cross-metathesis as the key reaction [28, 29], which will enable the divergent and efficient synthesis of chlamydocin-type structural analogs through the introduction of various side chains. This strategy would allow us to access natural cyclic peptides such as koshidacin B (**1**), Ac-TAN-1746 (**2**), and TAN-1746 (**3**) from a common intermediate **4** and the corresponding terminal olefin fragments **5** via olefin cross-metathesis/reduction sequence. For the cyclic peptide intermediate **4**, we have chosen the less hindered -proline and -allylglycine as the cyclization sites. Further disconnection would provide us with the four amino acid fragments **7**, **8**, **9**, and **10**.

**Fig. 3:**
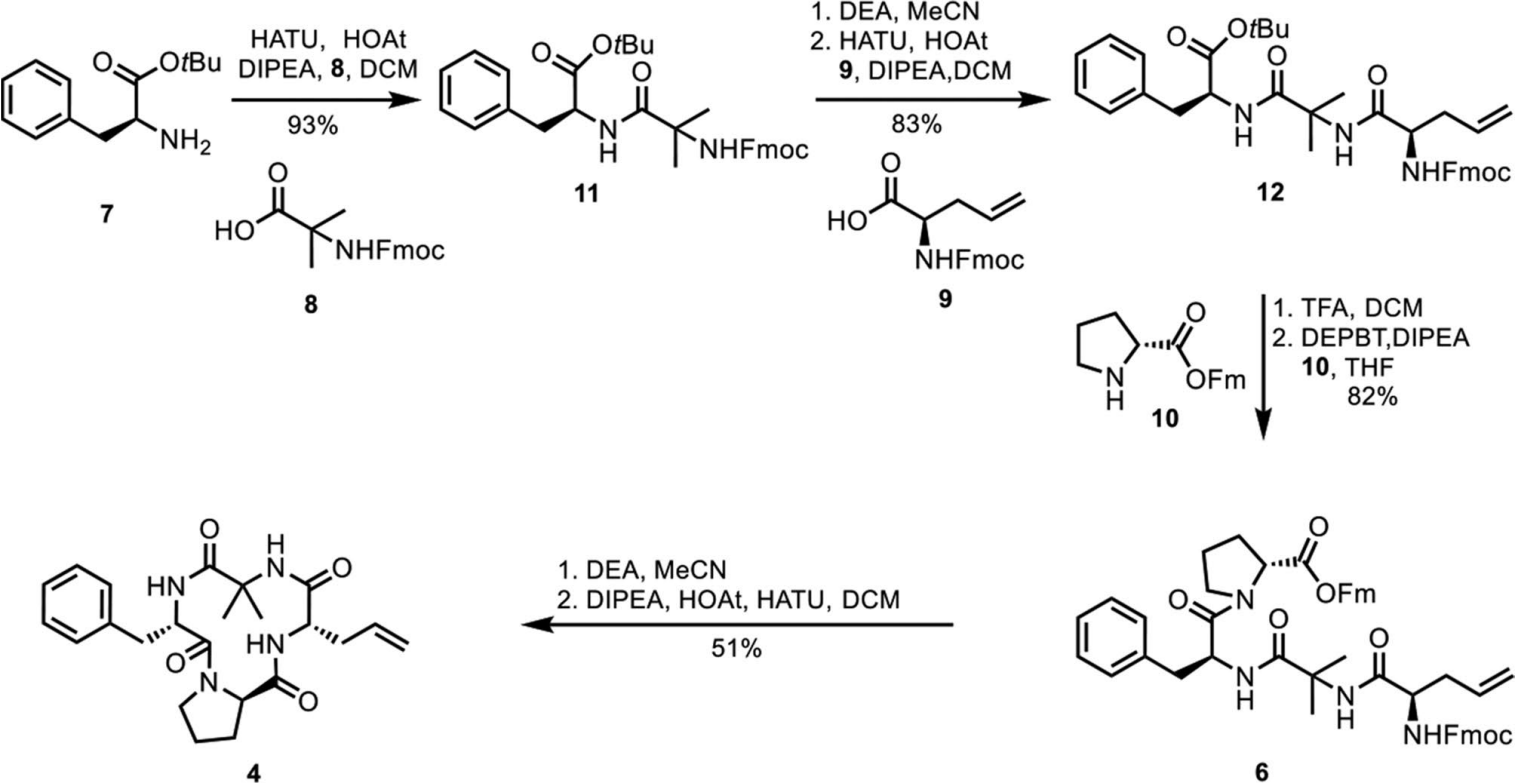
Synthesis of common intermediate **4**

As shown in Fig. 3, our total synthesis started with the construction of the common cyclic peptide intermediate **4**. Coupling of H-ʟ-Phe-O*t*Bu with acid **8** under HATU/HOAT/DIPEA conditions afforded the dipeptide compound **11** in a high yield of 93%. After removing the Fmoc protecting group with diethylamine, the deprotected dipeptide was coupled with acid **9** using HATU as the coupling reagent, affording the tripeptide fragment **12** in 83% overall yield. Removal of the *tert*-butyl ester with TFA, followed by peptide coupling with proline **10** under DEPBT/DIPEA/THF conditions, gave the tetrapeptide fragment **6** in 82% overall yield. Treatment with DEA, followed by macrocyclization under HATU/HOAT/DIPEA conditions, provided the desired cyclic amide product **4** in 51% overall yield.

**Fig. 4:**
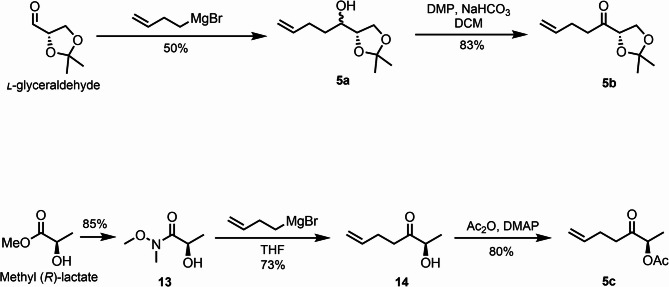
Synthesis of terminal olefin **5b** and **5c**

After securing the common cyclic peptide intermediate **4**, we commenced the synthesis of the various terminal olefin fragments **5** (**5b**, **5c**), as illustrated in Fig. 4. The known compound **5a** was accessible through the addition of allylmagnesium bromide to ʟ-glyceraldehyde in 50% yield. This compound was further oxidized using Dess-Martin periodinane to furnish the olefin compound **5b** in 83% yield. The synthesis of **5c** began with methyl (*R*)-lactate, which was first converted to a Weinreb amide **13** and then subjected to a Grignard addition with allylmagnesium bromide to provide the alcohol **14** in 73% yield. Subsequent acetylation of the alcohol furnished the terminal olefin compound **5c** in 80% yield.

With compounds **4** and **5** in hand, we proceeded to explore the olefin cross-metathesis (CM) reaction (Table 1). The CM reaction of subjects **4** and **5b** was initially explored using dichloromethane (DCM) as the solvent and Grubbs I and Grubbs II catalysts at ambient temperature, furnishing the desired product **6** in 15% and 36% yields, respectively (Table 1, entries 1–2). Subsequently, increasing the reaction temperature to 50 °C under sealed conditions led to an improved yield of 45% for **6** (Table 1, entry 3). Furthermore, the use of Hoveyda-Grubbs II catalyst in DCE at 50 °C provided **6** in 39% yield (Table 1, entry 4). In contrast, employing Hoveyda-Grubbs II in toluene at 80 °C led to a lower yield of 32% for **6** (Table 1, entry 5). Notably, the conditions reported by Lipshutz and co-workers, utilizing CuI in diethyl ether at 40 °C [30], afforded the desired product **6** in an improved 57% yield (Table 1, entry 6).

**Table 1:**
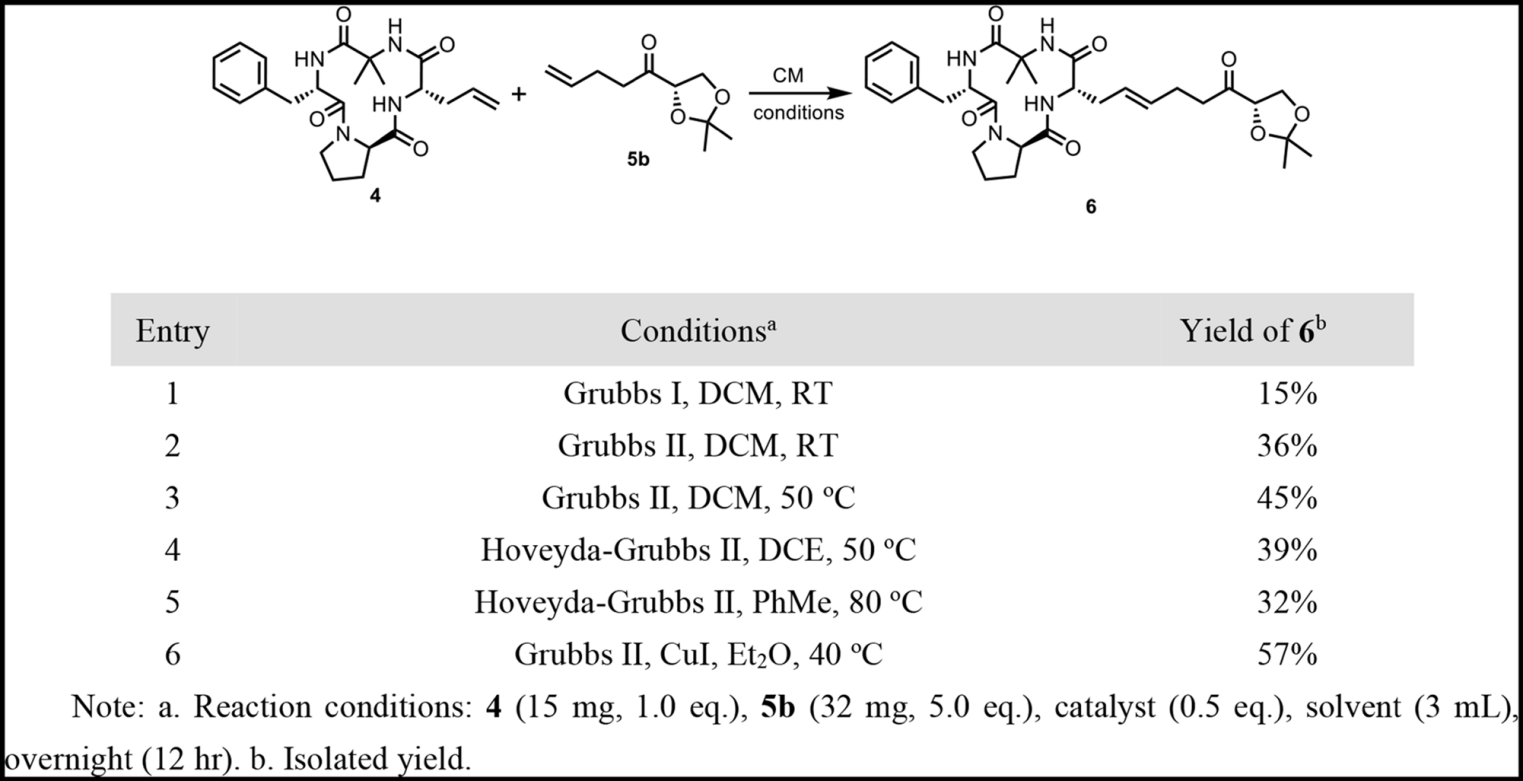
Screening of conditions for olefin cross-metathesis of Cyclic peptide **4** and olefin **5b**

**Fig. 5:**
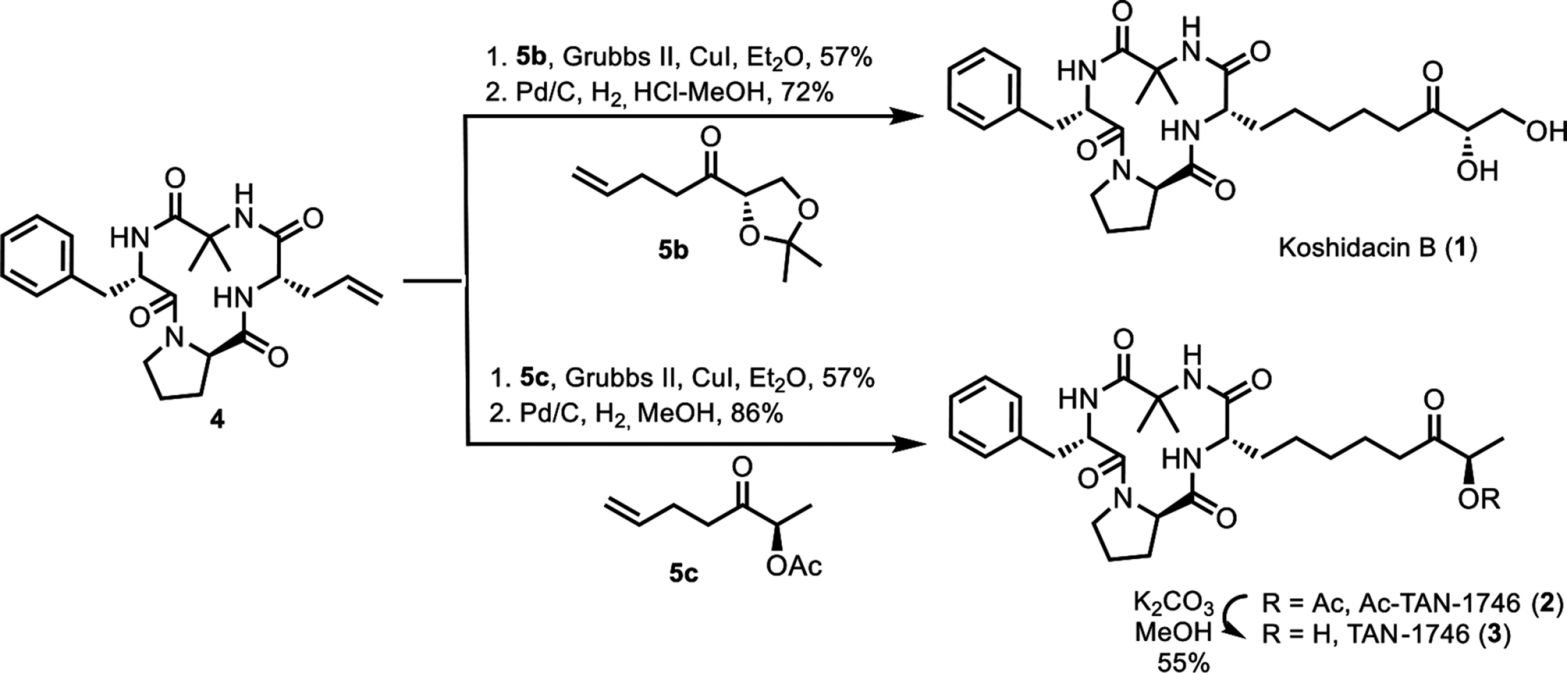
Total synthesis of natural occurring analogs of chlamydocin

Subsequently, hydrogenation of compound **6** over Pd/C under an H_2_ atmosphere, using HCl-methanol as the solvent, afforded koshidacin B (**1**) in 72% yield (Fig. 5). The analogous transformations of **4** with **5c** enabled the synthesis of the natural product Ac-TAN-1746 (**2**), which upon removal of the Ac- group, completed the total synthesis of TAN-1746 (**3**). The ^1^H NMR, ^13^C NMR data of our synthesized compounds koshidacin B, Ac-TAN-1746, and TAN-1746 are in complete agreement with the corresponding spectroscopic data of the natural products (see Table S1-3 in Supplementary Information). The optical rotations of the three synthetic compounds were in good agreement with those reported for the natural products: $\:{\left[{\upalpha\:}\right]}_{\text{D}}^{20}$= −80.0 (*c* 0.1, CHCl_3_) for synthetic koshidacin B, compared to $\:{\left[{\upalpha\:}\right]}_{\text{D}}^{25}$= −56.2 (*c* 0.1, CHCl_3_) [14] for the natural sample; $\:{\left[{\upalpha\:}\right]}_{\text{D}}^{20}$ = −48.0 (*c* 1.0, EtOH) for synthetic Ac-TAN-1746, compared to $\:{\left[{\upalpha\:}\right]}_{\text{D}}^{23}$= −45.7 (*c* 0.21, EtOH) [13] for the natural product; and $\:{\left[{\upalpha\:}\right]}_{\text{D}}^{20}$ = −71.1 (*c* 0.45, EtOH) for synthetic TAN-1746, compared to $\:{\left[{\upalpha\:}\right]}_{\text{D}}^{23}$= −51.0 (*c* 0.10, EtOH) [13] for the natural isolate.

**Fig. 6:**
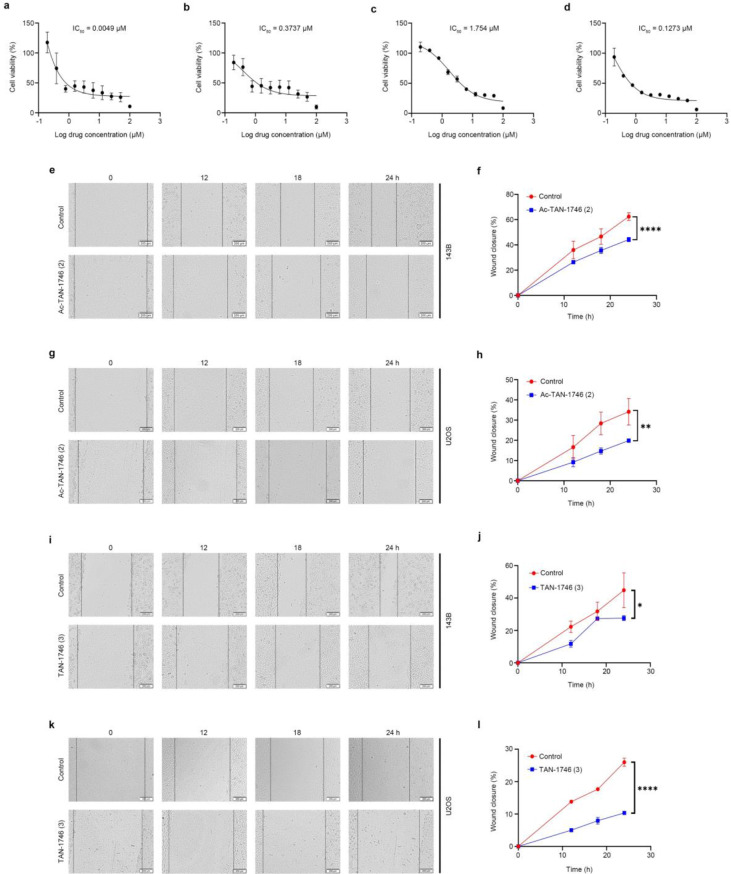
Ac-TAN-1746 (**2**) and TAN-1746 (**3**) effectively inhibits the proliferation and migration of osteosarcoma cells in vitro. (**a**, **b**) Ac-TAN-1746 (**2**) significantly reduced the cell viability of osteosarcoma cells. 143B (**a**) and U2OS (**b**) cells were treated with an increasing concentration gradient of Ac-TAN-1746 (**2**) for 72 h, and the cell viability was detected by CCK-8 assay. (**c**, **d**) TAN-1746 (**3**) also effectively decreased the cell viability of osteosarcoma cells. 143B (**c**) and U2OS (**d**) cells were treated with an increasing concentration gradient of TAN-1746 (**3**) for 72 h, and cell viability was measured by the CCK-8 assay. (**e**–**l**) Ac-TAN-1746 (**2**) and TAN-1746 (**3**) significantly inhibited the migration of osteosarcoma cells. 143B and U2OS cells were plated into 6-well plates, scratched with a 200 µL sterile pipette tip, and treated with 1 µM Ac-TAN-1746 or 2 µM TAN-1746 (**3**). The Wound closure was recorded by inverted fluorescence microscope at indicated time points. Representative images of each group and the quantitative results of the wound-healing assay are shown. Scale bar: 200 μm. Data are presented as mean ± SEM. *p < 0.05, **p < 0.01, ***p < 0.001, ****p < 0.0001, by student’s t test (**f**, **h**, **j** and **l**)

Having completed the total synthesis of koshidacin B (**1**), Ac-TAN-1746 (**2**), and TAN-1746 (**3**), we subsequently initiated their bioactivity screening (Fig. 6). Osteosarcoma, the most common primary malignant bone tumor, remains difficult to treat due to the development of drug resistance and the high toxicity of conventional chemotherapeutic agents. Accordingly, we evaluated the anti-osteosarcoma activities of these three synthetic natural products against the 143B and U2OS cell lines. Among the tested compounds, Ac-TAN-1746 (**2**) and TAN-1746 (**3**) demonstrated significant anti-osteosarcoma activity. The IC_50_ values of compound **2** were 4.9 nM against 143B cells and 0.37 µM against U2OS cells (Fig. 6a-b), while for compound **3** the IC_50_ values were 1.75 µM against 143B cells and 0.13 µM against U2OS cells (Fig. 6c-d). These effects were significantly superior to that of cisplatin. In contrast, koshidacin B (**1**) displayed minimal anti-osteosarcoma activity. Recognizing that metastasis is a hallmark of osteosarcoma aggressiveness, we further assessed the antimetastatic effects of Ac-TAN-1746 (**2**) and TAN-1746 (**3**) using a wound-healing assay. The 143B or U2OS cells were seeded in 6-well plate and grown to 90% confluence. And then, the cells were scratched using a 200-µL pipette tip and cultured with DMEM containing 1 µM Ac-TAN-1746 (**2**) or 2 µM TAN-1746 (**3**). The results showed that treatment with these compounds significantly inhibited the migration of 143B and U2OS cells (Fig. 6e-l). Taken together, these findings suggest that TAN-1746 and Ac-TAN-1746 possess both antiproliferative and antimetastatic properties against osteosarcoma cells in vitro.

## Experimental

### General

All reagents and anhydrous solvents were used as received unless otherwise noted. NMR spectra were recorded on 400, 500, or 600 MHz Bruker Avance instruments (Germany). HRMS data were acquired using a Thermo UltiMate 3000 UHPLC coupled with a Q-Exactive Orbitrap. Optical rotations were measured on a Rudolph AutoPol-I polarimeter (589 nm, 50 mm cell).

### Synthesis of *tert*-butyl (2-((((9 H-fluoren-9-yl)methoxy)carbonyl)amino)-2-methylpropanoyl)-L-phenylalaninate (11)

Carboxylic acid **8** (423.0 mg, 1.3 mmol) was taken up in DCM (20.0 mL) at 0 ^o^C, HOAt (353.9 mg, 2.6 mmol), DIPEA (2.4 mL, 13.7 mmol), amine **7** (500.0 mg, 2.0 mmol), and HATU (988.6 mg, 2.0 mmol) were added sequentially. Stirring was continued overnight at RT (room temperature). Upon completion of the reaction, saturated NH_4_Cl (aq.) was added to quench the mixture which was extracted with DCM, and washed with saturated NH_4_Cl (aq.) followed by brine. Organic solvents were combined and dried using anhydrous sodium sulfate, then filtered and evaporated under vacuum. The resulting residue was subjected to purification through silica gel flash chromatography (ethyl acetate/petroleum ether: 1/1) to afford dipeptide **11** (639.2 mg, 93% yield) as a white foamy solid. $\:{\left[{\upalpha\:}\right]}_{\text{D}}^{20}$ = +24.0 (*c* 1.0, CHCl_3_); HRMS (ESI, m/z) for C_32_H_37_N_2_O_5_^+^ [M + H]^+^: Calcd. 551.2516, found: 551.2523; ^1^H NMR (400 MHz, CDCl_3_) δ 7.83–7.75 (m, 2 H), 7.65–7.58 (m, 2 H), 7.46–7.39 (m, 2 H), 7.38–7.32 (m, 2 H), 7.29–7.20 (m, 3 H), 7.20–7.14 (m, 2 H), 6.63 (d, *J* = 7.5 Hz, 1H), 5.48 (s, 1H), 4.74 (d, *J* = 6.8 Hz, 1H), 4.51–4.33 (m, 2 H), 4.22 (t, *J* = 6.9 Hz, 1H), 3.13 (d, *J* = 6.0 Hz, 2 H), 1.59–1.46 (m, 6 H), 1.43 (s, 9 H); ^13^C NMR (101 MHz, CDCl_3_) δ 173.7, 170.5, 154.9, 143.9, 141.3, 136.2, 129.6, 128.3, 127.7, 127.1, 127.0, 125.1, 125.1, 120.0, 82.4, 66.7, 56.8, 53.7, 47.2, 37.9, 28.0, 25.5.

### Synthesis of *tert*-butyl (2-((R)-2-((((9 H-fluoren-9-yl)methoxy)carbonyl)amino)pent-4-enamido)-2-methylpropanoyl)-L-phenylalaninate (12)

Dipeptide **11** (317.2 mg, 0.60 mmol) was taken up in MeCN (5.0 mL), and DEA (diethylamine, 1.0 mL) was added at RT. The reaction mixture was stirred for 1.0 h at RT. Upon completion of the reaction, the solution was concentrated in vacuum. Following evaporation of DEA, the residue was dissolved in DCM (10.0 mL). HOAt (163.3 mg, 1.20 mmol), DIPEA (1.10 mL, 6.30 mmol), compound **9** (303.6 mg, 0.90 mmol), and HATU (456.3 mg, 1.20 mmol) were added to the solution at 0 °C, and stirring was continued overnight at RT. Upon completion of the reaction, saturated NH_4_Cl (aq.) was added to quench the mixture which was extracted with DCM, and washed with saturated NH_4_Cl (aq.) followed by brine. Organic solvents were combined and dried using anhydrous sodium sulfate, then filtered and evaporated under vacuum. The resulting residue was subjected to purification through silica gel flash chromatography (ethyl acetate/hexane: 1/2) to afford tripeptide **12** (311.6 mg, 83%) as a yellow foamy solid. $\:{\left[{\upalpha\:}\right]}_{\text{D}}^{20}$ = +12.0 (*c* 1.0, CHCl_3_); HRMS (ESI, m/z) for C_37_H_43_N_3_O_6_Na^+^ [M + Na]^+^: Calcd. 648.3044, found: 648.3055; ^1^H NMR (500 MHz, CDCl_3_) δ 7.84–7.74 (m, 2 H), 7.60 (dd, *J* = 7.5, 4.4 Hz, 2 H), 7.42 (t, *J* = 7.5 Hz, 2 H), 7.35–7.30 (m, 2 H), 7.30–7.24 (m, 2 H), 7.24–7.19 (m, 1H), 7.19–7.14 (m, 2 H), 6.76 (s, 1H), 6.69 (d, *J* = 7.4 Hz, 1H), 5.87–5.64 (m, 1H), 5.43 (d, *J* = 7.6 Hz, 1H), 5.16 (d, *J* = 11.3 Hz, 2 H), 4.72 (q, *J* = 6.1 Hz, 1H), 4.53–4.41 (m, 1H), 4.41–4.31 (m, 1H), 4.24 (t, *J* = 7.0 Hz, 1H), 4.21–4.11 (m, 1H), 3.11 (d, *J* = 6.2 Hz, 2 H), 2.62–2.42 (m, 2 H), 1.55 (s, 3 H), 1.51 (s, 3 H), 1.42 (s, 9 H); ^13^C NMR (126 MHz, CDCl_3_) δ 173.5, 170.5, 170.3, 156.1, 143.8, 143.7, 141.3, 136.2, 132.8, 129.6, 128.3, 127.8, 127.1, 127.0, 125.1, 120.0, 119.3, 82.4, 67.2, 57.3, 54.5, 53.8, 47.1, 38.6, 37.9, 28.0, 25.2, 24.7.

### Synthesis of (9 H-fluoren-9-yl)methyl (2-((R)-2-((((9 H-fluoren-9-yl)methoxy)carbonyl)amino)pent-4-enamido)-2-methylpropanoyl)-L-phenylalanyl-D-prolinate (6)

Tripeptide **12** (375.5 mg, 0.60 mmol) was taken up in DCM (10.0 mL), and TFA (2.0 mL) was added at RT, and stirring was continued for 1.5 h. Upon completion of the reaction, the solution was concentrated in vacuum. Following evaporation of TFA, the residue was dissolved in THF (10.0 mL). DIPEA (1.50 mL, 8.40 mmol), amine **10** (360.4 mg, 1.20 mmol), and DEPBT (478.6 mg, 1.60 mmol) were added to the solution, and stirring was continued overnight at RT. Upon completion of the reaction, saturated NH_4_Cl (aq.) was added to quench the mixture which was extracted with ethyl acetate, and washed with saturated NH_4_Cl (aq.) followed by brine. Organic solvents were combined and dried using anhydrous sodium sulfate, then filtered and evaporated under vacuum. The resulting residue was subjected to purification through silica gel flash chromatography (ethyl acetate/petroleum ether: 1/2) to afford tetrapeptide **6** (415.4 mg, 82%) as a yellow foamy solid. $\:{\left[{\upalpha\:}\right]}_{\text{D}}^{20}$ = +24.0 (*c* 1.0, CHCl_3_); HRMS (ESI, m/z) for C_52_H_52_N_4_O_7_Na^+^ [M + Na]^+^: Calcd. 867.3728, found: 867.3732; ^1^H NMR (500 MHz, CDCl_3_) δ 7.85–7.72 (m, 4 H), 7.67–7.51 (m, 4 H), 7.47–7.36 (m, 4 H), 7.36–7.18 (m, 8 H), 7.16–7.03 (m, 1H), 7.02–6.91 (m, 1H), 6.80 (s, 1H), 5.85–5.67 (m, 1H), 5.60–5.50 (m, 1H), 5.23–5.10 (m, 3 H), 4.97–4.86 (m, 1H), 4.58–4.49 (m, 1H), 4.49–4.42 (m, 1H), 4.42–4.38 (m, 1H), 4.38–4.30 (m, 1H), 4.21 (dd, *J* = 9.6, 6.4 Hz, 3 H), 3.56–3.42 (m, 1H), 3.10–3.01 (m, 1H), 3.01–2.92 (m, 1H), 2.74–2.64 (m, 1H), 2.62–2.53 (m, 1H), 2.53–2.41 (m, 1H), 1.85–1.79 (m, 1H), 1.62–1.56 (m, 1H), 1.54–1.45 (m, 2 H), 1.53 (s, 3 H), 1.48 (s, 3 H); ^13^C NMR (126 MHz, CDCl_3_) δ 173.5, 171.6, 170.2, 169.8, 144.0, 143.4, 141.4, 141.4, 129.6, 128.5, 127.9, 127.8, 127.2, 127.1, 127.1, 125.2, 125.1, 125.1, 120.0, 67.2, 66.5, 58.9, 57.3, 52.7, 47.2, 47.0, 46.8, 39.4, 36.9, 28.9, 25.4, 24.3.

### Synthesis of (*3 S*,*9 S*,*14*a*R*)-3-allyl-9-benzyl-6,6-dimethyldecahydropyrrolo[1,2-a][1,4,7,10]tetraazacyclo-dodecine-1,4,7,10-tetraone (4)

Tetrapeptide **6** (498.6 mg, 0.59 mmol) was taken up in MeCN (5.0 mL), and DEA (1.0 mL) was added at RT, and stirring was continued for 1.0 h. Upon completion, the solution was concentrated in vacuum. Following evaporation of DEA, the residue was dissolved in DCM (250.0 mL). The resulting solution was then added dropwise a DCM (10.0 mL) solution of HOAt (160.61 mg, 1.18 mmol), DIPEA (1.60 mL, 12.39 mmol), and HATU (448.67 mg, 1.18 mmol) by a syringe pump over 6 h at 0 °C. The reaction was maintained under stirring for 2 days and it was quenched with saturated NH_4_Cl (aq.), extracted with DCM, and washed with saturated NH_4_Cl (aq.) followed by brine. Organic solvents were combined and dried using anhydrous sodium sulfate, then filtered and evaporated under vacuum. The resulting residue was subjected to purification through silica gel flash chromatography (ethyl acetate/petroleum ether: 1/1) to afford cyclopeptide **4** (128.3 mg, 51%) as a white foamy solid. The spectroscopic data of compound **4** agree with those reported by Chen et al. [26]: $\:{\left[{\upalpha\:}\right]}_{\text{D}}^{20}$ = −108.0 (*c* 1.0, CHCl_3_); HRMS (ESI, m/z) for C_23_H_30_N_4_O_4_Na^+^ [M + Na]^+^: Calcd.449.2159, found: 449.2166; ^1^H NMR (500 MHz, CDCl_3_) δ 7.51 (d, *J* = 10.2 Hz, 1H), 7.34–7.20 (m, 5 H), 7.18 (d, *J* = 10.4 Hz, 1H), 6.05 (s, 1H), 5.75 (ddt, *J* = 17.1, 10.2, 6.8 Hz, 1H), 5.23–5.15 (m, 2 H), 5.12 (dt, *J* = 10.3, 1.4 Hz, 1H), 4.71–4.66 (m, 1H), 4.31 (dt, *J* = 10.3, 7.6 Hz, 1H), 3.88 (ddd, *J* = 10.1, 8.4, 4.5 Hz, 1H), 3.33–3.25 (m, 1H), 3.25–3.21 (m, 1H), 2.98 (dd, *J* = 13.5, 5.7 Hz, 1H), 2.64–2.56 (m, 1H), 2.45–2.37 (m, 1H), 2.37–2.30 (m, 1H), 2.25–2.14 (m, 1H), 1.83–1.75 (m, 2 H), 1.79 (s, 3 H), 1.36 (s, 3 H); ^13^C NMR (126 MHz, CDCl_3_) δ 175.6, 173.9, 172.9, 171.8, 137.0, 132.8, 129.1, 128.6, 126.7, 118.4, 58.8, 57.8, 53.9, 53.5, 47.0, 35.8, 33.3, 26.5, 25.0, 24.8, 23.5.

### Synthesis of (*S*)-1-(2,2-dimethyl-1,3-dioxolan-4-yl)pent-4-en-1-one (5b)

ʟ-glyceraldehyde (0.54 mL, 7.57 mmol) was taken up in THF (10.0 mL) at 0 °C, and 3-butenylmagnesium bromide (30.0 mL, 0.5 M in THF, 15.0 mmol) was added dropwise. Upon completion of the reaction, saturated NH_4_Cl (aq.) was added to quench the mixture which was extracted with ethyl acetate, and washed with saturated NH_4_Cl (aq.) followed by brine. Organic solvents were combined and dried using anhydrous sodium sulfate, then filtered and evaporated under vacuum. The resulting residue was subjected to purification through silica gel flash chromatography (ethyl acetate/petroleum ether: 1/5) to afford alcohol **5a** (0.72 g, 50.0%) as a colorless oil.

Alcohol **5a** (1.0 g, 5.4 mmol) was taken up in DCM (10.0 mL), sodium bicarbonate (1.36 g, 16.2 mmol) and Dess-Martin periodinane (DMP) (3.44 g, 8.1 mmol) were added at 0 °C. Stirring was continued at RT. After the reaction was complete, the mixture was filtered, and concentrated under reduced pressure, and the resulting residue was subjected to purification through silica gel flash chromatography (ethyl acetate/petroleum ether: 1/10) to afford ketone **5b** (0.82 g, 83%) as a colorless oil.

$\:{\left[{\upalpha\:}\right]}_{\text{D}}^{20}$ = −60.0 (*c* 1.0, CHCl_3_); HRMS (ESI, m/z) for C_10_H_16_O_3_Na^+^ [M + Na]^+^: Calcd.207.0992, found: 207.0996; ^1^H NMR (400 MHz, CDCl_3_) δ 5.91–5.74 (m, 1H), 5.11–4.92 (m, 2 H), 4.49–4.39 (m, 1H), 4.24–4.13 (m, 1H), 4.01–3.91 (m, 1H), 2.78–2.63 (m, 2 H), 2.40–2.22 (m, 2 H), 1.48 (s, 3 H), 1.39 (s, 3 H); ^13^C NMR (126 MHz, CDCl_3_) δ 210.1, 136.9, 115.3, 110.9, 80.2, 66.5, 37.7, 26.9, 26.0, 25.0.

### Synthesis of Koshidacin B

To a flame-dried 35 mL sealed tube containing cyclopeptide **4** (100.0 mg, 0.235 mmol) was added Grubbs 2nd generation catalyst (101.9 mg, 0.117 mmol), **5b** (221.0 mg, 1.175 mmol) and CuI (4.6 mg, 0.024 mmol) under an N_2_ atmosphere. Freshly distilled ethyl ether (5.0 mL) was added and the mixture was gradually brought to 40 °C (oil bath temperature) overnight. After cooling to RT, the reaction mixture was concentrated in vacuum, and the resulting residue was subjected to purification through silica gel flash chromatography (ethyl acetate/petroleum ether: 1/1) to afford ketone **6** (80.2 mg, 57%) as a white foamy solid. $\:{\left[{\upalpha\:}\right]}_{\text{D}}^{20}$ = −95.0 (*c* 0.8, CHCl_3_); HRMS (ESI, m/z) for C_31_H_42_N_4_O_7_Na^+^ [M + Na]^+^: Calcd.605.2946, found: 605.2950; ^1^H NMR (500 MHz, CDCl_3_) δ 7.52 (d, *J* = 10.1 Hz, 1H), 7.33–7.20 (m, 5 H), 7.15 (d, *J* = 10.3 Hz, 1H), 6.10 (s, 1H), 5.56 (dt, *J* = 14.1, 6.7 Hz, 1H), 5.42–5.30 (m, 1H), 5.18 (td, *J* = 10.1, 5.7 Hz, 1H), 4.72–4.66 (m, 1H), 4.43 (dd, *J* = 7.8, 5.5 Hz, 1H), 4.28–4.17 (m, 2 H), 4.05–3.91 (m, 1H), 3.92–3.81 (m, 1H), 3.31–3.20 (m, 2 H), 2.97 (dd, *J* = 13.5, 5.7 Hz, 1H), 2.74–2.61 (m, 2 H), 2.54–2.48 (m, 1H), 2.44–2.23 (m, 5 H), 2.22–2.14 (m, 1H), 1.84–1.70 (m, 1H), 1.79 (s, 3 H), 1.50 (s, 3 H), 1.41 (s, 3 H), 1.36 (s, 3 H); ^13^C NMR (151 MHz, CDCl_3_) δ 210.1, 175.6, 173.9, 172.8, 171.8, 137.0, 132.6, 129.1, 128.6, 126.7, 125.3, 111.0, 80.2, 66.5, 58.8, 57.8, 54.3, 53.5, 47.0, 38.2, 35.8, 32.1, 26.4, 26.1, 25.9, 25.0, 25.0, 24.7, 23.6.

Ketone **6** (15.0 mg, 25.8 µmol) was taken up in 0.5 mL of methanol hydrochloride (prepared by adding 7.2 µL of SOCl_2_ to 2.0 mL of MeOH, and using 0.5 mL of the resulting solution), and 5% Pd/C (3.0 mg) was added at RT. Stirring was continued overnight at RT under an H_2_ atmosphere. After filtration, the solvent was removed under reduced pressure, and the resulting residue was subjected to purification through silica gel flash chromatography (ethyl acetate/petroleum ether: 1/1) to afford koshidacin B (10.1 mg, 72%) as a white foamy solid. $\:{\left[{\upalpha\:}\right]}_{\text{D}}^{20}$ = −80.0 (*c* 0.1, CHCl_3_){$\:{\left[{\upalpha\:}\right]}_{\text{D}}^{25}$ = −56.2 (*c* 0.1, CHCl_3_) [14] for natural koshidacin B}; HRMS (ESI, m/z) for C_28_H_41_N_4_O_7_^+^ [M + H]^+^: Calcd.545.2970, found: 545.2979; ^1^H NMR (600 MHz, CDCl_3_) δ 7.48 (d, *J* = 10.3 Hz, 1H), 7.33–7.19 (m, 5 H), 7.15 (d, *J* = 10.0 Hz, 1H), 6.10 (s, 1H), 5.16 (ddd, *J* = 10.1, 5.7 Hz, 1H), 4.72–4.61 (m, 1H), 4.21 (t, *J* = 3.2 Hz, 1H), 4.20–4.14 (m, 1H), 4.00–3.90 (m, 2 H), 3.89–3.82 (m, 1H), 3.29–3.24 (m, 1H), 3.24–3.18 (m, 1H), 2.95 (dd, *J* = 13.5, 5.7 Hz, 1H), 2.67–2.57 (m, 1H), 2.57–2.48 (m, 1H), 2.38–2.26 (m, 1H), 2.20–2.11 (m, 1H), 1.86–1.68 (m, 3 H), 1.77 (s, 3 H), 1.65–1.49 (m, 3 H), 1.34 (s, 3 H), 1.38–1.24 (m, 4 H); ^13^C NMR (151 MHz, CDCl_3_) δ 209.8, 175.6, 174.3, 172.9, 171.9, 137.0, 129.0, 128.6, 126.8, 77.5, 63.7, 58.8, 57.8, 54.2, 53.4, 47.0, 37.5, 35.8, 28.7, 28.6, 26.4, 25.0, 24.9, 24.8, 23.6, 22.7.

### Synthesis of (*S*)-3-oxohept-6-en-2-yl acetate (5c)

(+)-Methyl D-lactate (0.92 mL, 9.61 mmol) and *N*,*O*-dimethylhydroxylamine hydrochloride (2.34 g, 24.03 mmol) were taken up in THF (60.0 mL), and *i*-PrMgCl (48.0 mL, 1.0 M in THF, 48.00 mmol) was added at − 20 °C over 30 min. Stirring was continued for another 30 min at 0 °C and was quenched with saturated NH_4_Cl (aq.) and extracted with ethyl acetate. Organic solvents were combined and dried using anhydrous sodium sulfate, then filtered and evaporated under vacuum. The resulting residue was subjected to purification through silica gel flash chromatography (ethyl acetate/petroleum ether: 1/1) to afford Weinreb amide **13** (1.1 g, 85%) as a slightly yellow oil.$\:{\:\left[{\upalpha\:}\right]}_{\text{D}}^{20}$ = +44.0 (*c* 1.0, CHCl_3_); HRMS (ESI, m/z) for C_5_H_11_NO_3_Na^+^ [M + Na]^+^: Calcd.156.0631, found: 156.0641; ^1^H NMR (500 MHz, CDCl_3_) δ 4.48 (p, *J* = 6.7 Hz, 1H), 3.71 (s, 3 H), 3.43 (d, *J* = 7.9 Hz, 1H), 3.23 (s, 3 H), 1.35 (d, *J* = 6.6 Hz, 3 H); ^13^C NMR (126 MHz, CDCl_3_) δ 175.7, 65.0, 61.3, 32.4, 21.0.

Weinreb amide **13** (500.0 mg, 3.76 mmol) was taken up in THF (10.0 mL) at 0 °C, and 3-butenylmagnesium bromide (15.0 mL, 0.5 M in THF, 7.5 mmol) was added dropwise. Upon completion of the reaction, saturated NH_4_Cl (aq.) was added to quench the mixture followed by extracted with ethyl acetate, washed with saturated NH_4_Cl (aq.), and brine. Organic solvents were combined and dried using anhydrous sodium sulfate, then filtered and evaporated under vacuum. The resulting residue was subjected to purification through silica gel flash chromatography (ethyl acetate/petroleum ether: 1/5) to afford alcohol **14** (351.6 mg, 73%) as a colorless oil.$\:\:{\left[{\upalpha\:}\right]}_{\text{D}}^{20}$ = −36.0 (*c* 1.0, CHCl_3_); HRMS (ESI, m/z) for C_7_H_12_O_2_Na^+^ [M + Na]^+^: Calcd.151.0730, found: 151.0736; ^1^H NMR (500 MHz, CDCl_3_) δ 5.90–5.70 (m, 1H), 5.14–4.87 (m, 2 H), 4.32–4.14 (m, 1H), 3.60 (d, *J* = 4.6 Hz, 1H), 2.68–2.58 (m, 1H), 2.57–2.49 (m, 1H), 2.46–2.26 (m, 2 H), 1.39 (d, 3 H); ^13^C NMR (126 MHz, CDCl_3_) δ 211.8, 136.5, 115.7, 72.7, 36.7, 27.4, 19.8.

Alcohol **14** (500.0 mg, 3.98 mmol) was taken up in acetic anhydride (10.0 mL) at 0 °C, and DMAP (4-(dimethylamino)pyridine, 433.2 mg, 1.95 mmol) was added. Stirring was continued at RT. Upon completion of the reaction, the mixture was quenched with 1.0 M aqueous HCl, extracted with dichloromethane, and washed with saturated NaHCO_3_ (aq.) followed by brine. Organic solvents were combined and dried using anhydrous sodium sulfate, then filtered and evaporated under vacuum. The resulting residue was subjected to purification through silica gel flash chromatography (ethyl acetate/petroleum ether: 1/5) to afford acetate **5c** (530.2 mg, 80%) as a colorless oil. $\:{\left[{\upalpha\:}\right]}_{\text{D}}^{20}$ = +28.0 (*c* 1.0, CHCl_3_); HRMS (ESI, m/z) for C_9_H_14_O_3_Na^+^ [M + Na]^+^: Calcd.193.0835, found: 193.0843; ^1^H NMR (500 MHz, CDCl_3_) δ 5.90–5.75 (m, 1H), 5.17–5.08 (m, 1H), 5.05–4.97 (m, 2 H), 2.71–2.61 (m, 1H), 2.59–2.49 (m, 1H), 2.43–2.27 (m, 2 H), 2.15 (s, 3 H), 1.41 (d, *J* = 7.0 Hz, 3 H); ^13^C NMR (126 MHz, CDCl_3_) δ 206.9, 170.4, 136.8, 115.5, 74.6, 37.4, 27.1, 20.8, 16.1.

### Synthesis of Ac-TAN-1746

To a flame-dried 35 mL sealed tube containing cyclopeptide **4** (26.0 mg, 0.061 mmol), Grubbs 2nd generation catalyst (26.3 mg, 0.031 mmol), compound **5c** (51.8 mg, 0.305 mmol), and CuI (1.2 mg, 0.0061 mmol) was added under an N_2_ atmosphere. Freshly distilled ethyl ether (5.0 mL) was added, and the solution was heated at 40 °C (oil bath temperature) overnight. After cooling to RT, the reaction mixture was concentrated in vacuo, and the resulting residue was subjected to purification through silica gel flash chromatography (ethyl acetate/petroleum ether: 1/1) to afford ketone **7** (19.8 mg, 57%) as a colorless oil.$\:\:{\left[{\upalpha\:}\right]}_{\text{D}}^{20}$ = −40.0 (*c* 0.6, CHCl_3_); HRMS (ESI, m/z) for C_30_H_40_N_4_O_7_Na^+^ [M + Na]^+^: Calcd.591.2789, found: 591.2798; ^1^H NMR (500 MHz, CDCl_3_) δ 7.53 (d, *J* = 10.3 Hz, 1H), 7.33–7.18 (m, 5 H), 7.14 (d, *J* = 10.3, 1H), 6.07 (s, 1H), 5.60–5.43 (m, 1H), 5.43–5.28 (m, 1H), 5.18 (td, *J* = 10.1, 5.7 Hz, 1H), 5.09 (q, *J* = 7.1 Hz, 1H), 4.71–4.63 (m, 1H), 4.31–4.18 (m, 1H), 3.94–3.80 (m, 1H), 3.33–3.19 (m, 2 H), 2.97 (dd, *J* = 13.5, 5.7 Hz, 1H), 2.66–2.42 (m, 3 H), 2.42–2.25 (m, 4 H), 2.25–2.13 (m, 1H), 2.15 (s, 3 H), 1.83–1.71 (m, 2 H), 1.78 (s, 3 H), 1.40 (d, *J* = 7.1 Hz, 3 H), 1.36 (s, 3 H); ^13^C NMR (126 MHz, CDCl_3_) δ 206.8, 175.6, 174.0, 172.8, 171.8, 170.4, 137.0, 132.3, 129.0, 128.6, 126.7, 125.5, 74.7, 58.8, 57.8, 54.2, 53.4, 47.0, 37.8, 35.8, 32.1, 26.4, 26.2, 25.0, 24.7, 23.6, 20.8, 16.1.

Ketone **7** (14.0 mg, 0.025 mmol) was taken up in 0.5 mL of MeOH, and 5% Pd/C (3.0 mg) was added at RT. Stirring was continued overnight at RT under an H_2_ atmosphere. After filtration, the filtrate was concentrated under reduced pressure, and the resulting residue was subjected to purification through silica gel flash chromatography (ethyl acetate/petroleum ether: 1/1) to afford Ac-TAN-1746 (12.3 mg, 86%) as a white foamy solid.

$\:{\left[{\upalpha\:}\right]}_{\text{D}}^{20}$ = −48.0 (*c* 1.0, EtOH) $\:\{{\left[{\upalpha\:}\right]}_{\text{D}}^{23}$ = −45.7 (*c* 0.21, EtOH) [13] for natural Ac-TAN-1746}; HRMS (ESI, m/z) for C_30_H_42_N_4_O_7_Na^+^ [M + Na]^+^: Calcd.593.2946, found: 593.2945; ^1^H NMR (500 MHz, CDCl_3_) δ 7.53 (d, *J* = 10.5 Hz, 1H), 7.30–7.25 (m, 2 H), 7.24–7.18 (m, 3 H), 7.11 (d, *J* = 10.2 Hz, 1H), 6.08 (s, 1H), 5.16 (ddd, *J* = 10.2, 10.2, 5.2 Hz, 1H), 5.08 (q, *J* = 7.0 Hz, 1H), 4.71–4.62 (m, 1H), 4.19 (ddd, *J* = 10.4, 10.4, 7.5 Hz, 1H), 3.85 (ddd, *J* = 9.7, 9.2, 4.4 Hz, 1H), 3.26 (dd, *J* = 13.5, 9.9 Hz, 1H), 3.23–3.16 (m, 1H), 2.95 (dd, *J* = 13.6, 5.7 Hz, 1H), 2.51 (ddd, *J* = 16.4, 7.2, 7.2 Hz, 1H), 2.40 (ddd, *J* = 17.5, 7.2, 7.2 Hz, 1H), 2.35–2.28 (m, 1H), 2.21–2.11 (m, 1H), 2.13 (s, 3 H), 1.83–1.71 (m, 3 H), 1.77 (s, 3 H), 1.66–1.54 (m, 3 H), 1.38 (d, *J* = 7.1 Hz, 3 H), 1.34 (s, 3 H), 1.33–1.24 (m, 4 H); ^13^C NMR (126 MHz, CDCl_3_) δ 207.6, 175.7, 174.4, 172.9, 171.9, 170.5, 137.0, 129.1, 128.6 126.7, 74.6, 58.7, 57.8, 54.3, 53.4, 46.9, 38.0, 35.8, 29.7, 28.8, 28.7, 26.4, 25.3, 25.0, 24.7, 23.6, 22.9, 20.8, 16.2.

### Synthesis of TAN-1746

Ac-TAN-1746 (12.0 mg, 0.02 mmol) was taken up in methanol (4.0 mL), and K_2_CO_3_ (8.8 mg, 0.06 mmol) was added at RT. Stirring was continued for 30 min at RT. Saturated aqueous NH₄Cl was added to quench the reaction, followed by extraction with ethyl acetate. The combined organic phases were washed with saturated NH_4_Cl (aq.) and brine. Organic solvents were combined and dried using anhydrous sodium sulfate, then filtered and evaporated under vacuum. The resulting residue was subjected to purification through silica gel flash chromatography (ethyl acetate/petroleum ether: 2:1) to afford TAN-1746 (6.0 mg, 55%) as a white foam.$\:\:{\left[{\upalpha\:}\right]}_{\text{D}}^{20}$ = −71.1 (*c* 0.45, EtOH) {$\:{\left[{\upalpha\:}\right]}_{\text{D}}^{23}$ = −51.0 (*c* 0.10, EtOH) [13] for natural TAN-1746}; HRMS (ESI, m/z) for C_28_H_41_N_4_O_6_^+^ [M + H]^+^: Calcd.529.3021, found: 529.3029; ^1^H NMR (500 MHz, CDCl_3_) δ 7.51 (d, *J* = 10.2 Hz, 1H), 7.29–7.25 (m, 2 H), 7.24–7.18 (m, 3 H), 7.13 (d, *J* = 10.3 Hz, 1H), 6.05 (s, 1H), 5.16 (ddd, *J* = 10.2, 10.2, 5.8 Hz, 1H), 4.68–4.64 (m, 1H), 4.23 (q, *J* = 7.5 Hz, 1H), 4.21–4.14 (m, 1H), 3.86 (ddd, *J* = 10.4, 8.4, 4.5 Hz, 1H), 3.26 (dd, *J* = 13.5, 10.0 Hz, 1H), 3.20 (ddd, *J* = 10.2, 7.4, 7.4 Hz, 1H), 2.95 (dd, *J* = 13.5, 5.7 Hz, 1H), 2.51 (ddd, *J* = 17.1, 7.3, 7.3 Hz, 1H), 2.42 (ddd, *J* = 17.1, 7.3, 7.3 Hz, 1H), 2.36–2.28 (m, 1H), 2.19–2.12 (m, 1H), 1.77 (s, 3 H), 1.85–1.70 (m, 3 H), 1.67–1.58 (m, 3 H), 1.38 (d, *J* = 7.1 Hz, 3 H), 1.34 (s, 3 H), 1.32–1.23 (m, 4 H); ^13^C NMR (126 MHz, CDCl_3_) δ 212.5, 175.6, 174.4, 172.9, 171.9, 137.0, 129.1, 128.6, 126.7, 72.6, 58.8, 57.8, 54.3, 53.4, 47.0, 37.3, 35.8, 28.8, 28.7, 26.4, 25.2, 25.0, 24.8, 23.6, 23.3, 19.9.

## Antiosteosarcoma activity assay in vitro

### Cell lines and cell culture

Human osteosarcoma cell line 143B were purchased from the American Type Culture Collection (ATCC; MD, USA), and U2OS cells were obtained from the Cell Bank of Chinese Academy of Sciences (Shanghai, China). They were cultured in DMEM (Cat #12800-58, gibco, USA) containing 10% fetal bovine serum (FBS, Cat# p30922, TransSerum, China), 2 mM L-glutamine, 0.1‰ penicillin (Cat# C15238072, Macklin, China), 0.1% streptomycin (Cat# C15108162, Macklin, China) at 37 °C in a humidified incubator with 5% CO_2_.

### Cell viability assay

Cell viability was measured by cell counting kit-8 (CCK-8, Cat# K1018, APExBIO, USA). 143B and U2OS cells were seeded in 96-well plates with total volume of 100 µL culture medium and cultured with increasing concentrations of compound for 72 h. CCK-8 (10 µL) was added 1 h before reading the absorbance at wavelength 450 nm.

### Wound healing assay

The ability of migration was detected by wound healing assay. Briefly, 143B and U2OS cells (8 × 10^5^ cells/well) were seeded in 6-well plates and grown to confluence, and then the cells were scratched with a 200 µL sterile pipette tip. Cells were washed with PBS to remove the cell fragment and replaced with fresh DMEM containing 2% FBS and 1 µM Ac-TAN-1746 (**2**) or 2 µM TAN-1746 (**3**). The Wound closure was recorded by inverted fluorescence microscope (Olympus, Japan) at 0 h, 12 h, 18 h, and 24 h [31].

## Conclusions

In summary, we achieved the efficient and convergent total syntheses of three naturally occurring chlamydocin analogs—koshidacin B, TAN-1746, and Ac-TAN-1746—each in a longest linear sequence of 9–10 steps. Based on this synthesis, the anti-osteosarcoma activities of these three chlamydocin analogs were evaluated. Among them, Ac-TAN-1746 (**2**), and TAN-1746 (**3**) demonstrated significant anti-osteosarcoma proliferation and migration activity in vitro. Beyond synthesizing these natural products, we are also working on generating diverse analogs by modifying side chain structures and incorporating various functional groups. This effort is currently underway in the laboratory.

## Electronic supplementary material

Below is the link to the electronic supplementary material.

Supplementary Material 1

## Data Availability

No datasets were generated or analysed during the current study.

## References

[1] Degenkolb T, Gams W, Brückner H. Natural cyclopeptaibiotics and related Cyclic tetrapeptides: structural diversity and future prospects. Chem Biodivers. 2008;5(5):693–706. 18493956 10.1002/cbdv.200890066

[2] Sarojini V, Cameron AJ, Varnava KG, Denny WA, Sanjayan G. Cyclic tetrapeptides from nature and design: A review of synthetic methodologies, structure, and function. Chem Rev. 2019;119(17):10318–59. 31418274 10.1021/acs.chemrev.8b00737

[3] Closse A, Huguenin R. Isolierung und strukturaufklärung von Chlamydocin. Helv Chim Acta. 1974;57(3):533–45. 4857466 10.1002/hlca.19740570306

[4] Walton JD, Earle ED, Gibson BW. Purification and structure of the host-specific toxin from helminthosporiumcarbonum race 1. Biochem Biophys Res Commun. 1982;107(3):785–94. 6890350 10.1016/0006-291x(82)90592-7

[5] Umehara K, Nakahara K, Kiyoto S, Iwami M, Okamoto M, Tanaka H, et al. Studies on WF-3161, a new antitumor antibiotic. J Antibiot. 1983;36(5):478–83. 10.7164/antibiotics.36.478 6860430

[6] Takayama S, Isogai A, Nakata M, Suzuki H, Suzuki A. Structure of Cyl-1, a novel cyclotetrapeptide from *Cylindrocladium scoparium*. Agric Biol Chem. 1984;48(3):839–42.

[7] Hirota A, Suzuki A, Suzuki H, Tamura S. Isolation and biological activity of Cyl-2, a metabolite of cylindrocladium scoparium. Agric Biol Chem. 1973;37(3):643–7.

[8] Itazaki H, Nagashima K, Sugita K, Yoshida H, Kawamura Y, Yasuda Y, et al. Isolation and structural Elucidation of new cyclotetrapeptides, Trapoxins A and B, having detransformation activities as antitumor agents. J Antibiot. 1990;43(12):1524–32. 10.7164/antibiotics.43.1524 2276972

[9] Newkirk TL, Bowers AA, Williams RM. Discovery, biological activity, synthesis and potential therapeutic utility of naturally occurring histone deacetylase inhibitors. Nat Prod Rep. 2009;26(10):1293–320. 19779641 10.1039/b817886k

[10] Mann BS, Johnson JR, Cohen MH, Justice R, Pazdur R. FDA approval summary: Vorinostat for treatment of advanced primary cutaneous T-Cell lymphoma. Oncologist. 2007;12(10):1247–52. 17962618 10.1634/theoncologist.12-10-1247

[11] Gupta S, Peiser G, Nakajima T, Hwang Y-S. Characterization of a phytotoxic cyclotetrapeptide, a novel Chlamydocin analogue, from verticillium coccosporum. Tetrahedron Lett. 1994;35(33):6009–12.

[12] Yoshimura K, Tsubotani S, Okazaki K. In., vol. 07–196686. Japan 1995.

[13] Tani H, Fujii Y, Nakajima H. Chlamydocin analogues from the soil fungus peniophora sp.: structures and plant growth-retardant activity. Phytochemistry. 2001;58(2):305–10. 11551555 10.1016/s0031-9422(01)00209-6

[14] Watanabe Y, Hachiya K, Ikeda A, Nonaka K, Higo M, Muramatsu R, et al. Koshidacins A and B, antiplasmodial Cyclic tetrapeptides from the Okinawan fungus Pochonia boninensis FKR-0564. J Nat Prod. 2022;85(11):2641–9. 36282784 10.1021/acs.jnatprod.2c00719

[15] Kawai M, Gardner JH, Rich DH. Stereoselective synthesis and absolute configuration of epoxyketones in Chlamydocin and related Cyclic tetrapeptides. Tetrahedron Lett. 1986;27(17):1877–80.

[16] Schmidt U, Lieberknecht A, Griesser H, Utz R, Beuttler T, Bartkowiak F. Amino acids and peptides; 55. synthesis of biologically active cyclopeptides; 7. Total synthesis of Chlamydocin. Synthesis. 1986;1986(05):361–6.

[17] Schmidt U, Lieberknecht A, Griesser H, Bartkowiak F. Stereoselective total synthesis of Chlamydocin and Dihydrochlamydocin. Angew Chem Int Ed. 1984;23(4):318–20.

[18] Baldwin JE, Adlington RM, Godfrey CRA, Patel VK. Stereospecific synthesis of Chlamydocin. Tetrahedron. 1993;49(36):7837–56.

[19] Quirin C, Kazmaier U. Synthesis of Chlamydocin by Chelate-Claisen rearrangement. Eur J Org Chem. 2009;2009(3):371–7.

[20] Elek GZ, Koppel K, Zubrytski DM, Konrad N, Järving I, Lopp M, Kananovich DG. Divergent access to histone deacetylase inhibitory cyclopeptides via a Late-Stage Cyclopropane ring cleavage strategy. Short synthesis of Chlamydocin. Org Lett. 2019;21(20):8473–8. 31596600 10.1021/acs.orglett.9b03305

[21] Nishino N, Kato T, Komatsu Y, Yoshida M. Design of Analogs of Trapoxin, Cyl-1, and Chlamydocin for MHC Class-I Molecule Up-Regulation. In: Lebl M, Houghten RA, editors. Peptides: The Wave of the Future: Proceedings of the Second International and the Seventeenth American Peptide Symposium, June 9–14, 2001, San Diego, California, USA. Dordrecht: Springer Netherlands; 2001. pp. 528-9.

[22] Bhuiyan MPI, Kato T, Okauchi T, Nishino N, Maeda S, Nishino TG, Yoshida M. Chlamydocin analogs bearing carbonyl group as possible ligand toward zinc atom in histone deacetylases. Bioorg Med Chem. 2006;14(10):3438–46. 16439135 10.1016/j.bmc.2005.12.063

[23] Wang S, Li X, Wei Y, Xiu Z, Nishino N. Discovery of potent HDAC inhibitors based on Chlamydocin with inhibitory effects on cell migration. ChemMedChem. 2014;9(3):627–37. 24285590 10.1002/cmdc.201300372

[24] Durand P, Peralba P, Derain V, Komesli S, Renaut P. Asymmetric total synthesis of natural diheteropeptin. Tetrahedron Lett. 2001;42(11):2121–4.

[25] Ghosh R, Biswas S, Bagchi A, Chattopadhyay SK. Synthesis and evaluation of 9-epi-Koshidacin B as selective inhibitor of histone deacetylase 1. J Nat Prod. 2024;87(12):2757–67. 39655856 10.1021/acs.jnatprod.4c00913

[26] Yang K, Tong Y, Li T, Sun Y, Wang L, Chen Y. Total syntheses of antimalarial Cyclic tetrapeptides: Koshidacins A and B via olefin Cross-Metathesis reactions. Synthesis. 2025;57(11):1875–84.

[27] Liu J, Long X-E, Nan H, Xing H, Liu H. In. China; 2024; NO. ZL2024112503895.

[28] Grubbs RH. Olefin-Metathesis catalysts for the Preparation of molecules and materials (Nobel Lecture). Angew Chem Int Ed. 2006;45(23):3760–5. 10.1002/anie.200600680 16724297

[29] Delaude L, Noels AF. Metathesis. In: Kirk-Othmer Encyclopedia of Chemical Technology. 2005.

[30] Voigtritter K, Ghorai S, Lipshutz BH. Rate enhanced olefin Cross-Metathesis reactions: the copper iodide effect. J Org Chem. 2011;76(11):4697–702. 21528868 10.1021/jo200360s PMC3352770

[31] Ye G, Jiao Y, Deng L, Cheng M, Wang S, Zhang J, et al. Beauvericin suppresses the proliferation and pulmonary metastasis of osteosarcoma by selectively inhibiting TGFBR2 pathway. Int J Biol Sci. 2023;19(14):4376–92. 37781043 10.7150/ijbs.86214 PMC10535710

