# Supplementary material for: Total synthesis and biological evaluation of Koshidacin B, TAN-1746, and Ac-TAN-1746
Source: BMC Chem. 2025 Jul 16;19(1):213. doi: 10.1186/s13065-025-01581-4 (PMC12265328; doi:10.1186/s13065-025-01581-4)
Supplement: Supplementary file 1 — Supplementary Material 1 [file 13065_2025_1581_MOESM1_ESM.pdf]

Supporting Information

Total synthesis and biological evaluation of koshidacin B, TAN-1746, and Ac-TAN-1746

Xiong-En Long[1†], Hailiang Xing[1†], Yixin He[2†], Xuanli Meng[2], Yanling Ma[1], Chang Liu[2], Xi Cao[1], Huiru Nan[1], Min-Jing Cheng[2*], Jia-Lei Yan[1*], Junyang Liu[1*]

[a]*School of Pharmacy and Food Engineering, Wuyi University, Jiangmen 529020, China*

[b]*Center for Bioactive Natural Molecules and Innovative Drugs, and Guangdong Province Key Laboratory of Pharmacodynamic Constituents of TCM and New Drugs Research, College of Pharmacy, Jinan University, Guangzhou 510632, China*

[†]These authors contributed equally to this work

*Correspondence: chengmj1235@jnu.edu.cn; yanjialei@wyu.edu.cn; liujy@wyu.edu.cn;

Table of Contents

1. Table S1. Comparison of ^{13}C NMR Data for Natural and Synthetic Kosidacin B in $CDCl_3$ S1

2. Table S2. Comparison of ^{13}C NMR Data for Natural and Synthetic TAN-1746 in $CDCl_3$ S2

3. Table S3. Comparison of ^{13}C NMR Data for Natural and Synthetic Ac-TAN-1746 in $CDCl_3$ S3

4. NMR Spectra S4

1. Table S1. Comparison of ^{13}C NMR Data for Natural and Synthetic Kosidacin B in $CDCl_3$

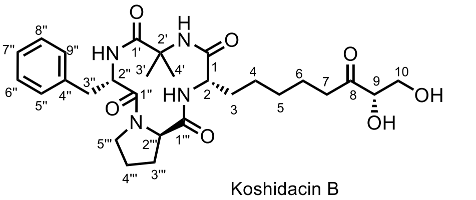

Koshidacin B

C No.	δC*i* (Natural)/ppm[a]	δC*s* (Synthetic)/ppm[b]	ΔδC (δC*s*-δC*i*)/ppm
**1**	174.3	174.3	0
**2**	54.3	54.2	-0.1
**3**	28.7	28.6	-0.1
**4**	25.0	25.0	0
**5**	28.5	28.7	0.2
**6**	22.8	22.7	-0.1
**7**	37.7	37.5	-0.2
**8**	210.0	209.8	-0.2
**9**	77.5	77.5	0
**10**	63.6	63.7	0.1
**1’**	175.6	175.6	0
**2’**	58.8	58.8	0
**3’**	23.5	23.6	0.1
**4’**	26.4	26.4	0
**1”**	172.9	172.9	0
**2”**	53.5	53.4	-0.1
**3”**	35.8	35.8	0
**4”**	137.0	137.0	0
**5”, 9”**	129.0	129.0	0
**6”, 8”**	128.6	128.6	0
**7”**	126.7	126.8	0.1
**1’”**	171.9	171.9	0
**2’”**	57.8	57.8	0
**3’”**	24.7	24.8	0.1
**4’”**	24.9	24.9	0
**5’”**	47.0	47.0	0

[a]Measured at 400 MHz; [b]Measured at 600 MHz.

2. Table S2. Comparison of ^{13}C NMR Data for Natural and Synthetic TAN-1746 in $CDCl_3$

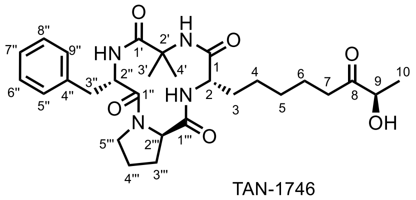

C No.	δC*i* (Natural)/ppm[a]	δC*s* (Synthetic)/ppm[b]	ΔδC (δC*s*-δC*i*)/ppm
**1**	174.3	174.4	0.1
**2**	54.3	54.3	0
**3**	28.74	28.8	0.06
**4**	25.2	25.2	0
**5**	28.69	28.7	0.01
**6**	23.2	23.3	0.1
**7**	37.2	37.3	0.1
**8**	212.4	212.5	0.1
**9**	72.6	72.6	0
**10**	19.8	19.9	0.1
**1’**	175.6	175.6	0
**2’**	58.8	58.8	0
**3’**	23.5	23.6	0.1
**4’**	26.4	26.4	0
**1”**	172.8	172.9	0.1
**2”**	53.4	53.4	0
**3”**	35.8	35.8	0
**4”**	137.0	137.0	0
**5”, 9”**	129.0	129.1	0.1
**6”, 8”**	128.6	128.6	0
**7”**	126.7	126.7	0
**1’”**	171.9	171.9	0
**2’”**	57.7	57.8	0.1
**3’”**	24.7	24.8	0.1
**4’”**	25.0	25.0	0
**5’”**	46.9	47.0	0.1

[a]Measured at 400 MHz; [b]Measured at 500 MHz.

3. Table S3. Comparison of ^{13}C NMR Data for Natural and Synthetic Ac-TAN-1746 in $CDCl_3$

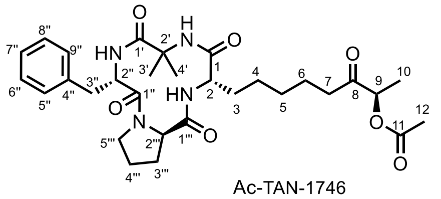

Ac-TAN-1746

C No.	δC*i* (Natural)/ppm[a]	δC*s* (Synthetic)/ppm[b]	ΔδC (δC*s*-δC*i*)/ppm
**1**	174.4	174.4	0
**2**	54.3	54.3	0
**3**	28.74	28.8	0.06
**4**	25.2	25.3	0.1
**5**	28.62	28.7	0.08
**6**	22.9	22.9	0
**7**	37.9	38.0	0.1
**8**	207.6	207.6	0
**9**	74.6	74.6	0
**10**	16.1	16.2	0.1
**11**	170.4	170.5	0.1
**12**	20.7	20.8	0.1
**1'**	175.6	175.7	0.1
**2'**	58.8	58.7	-0.1
**3'**	23.5	23.6	0.1
**4'**	26.4	26.4	0
**1"**	172.8	172.9	0.1
**2"**	53.4	53.4	0
**3"**	35.8	35.8	0
**4"**	137.0	137.0	0
**5", 9"**	129.0	129.1	0.1
**6", 8"**	128.6	128.6	0
**7"**	126.7	126.7	0
**1'''**	171.9	171.9	0
**2'''**	57.7	57.8	0.1
**3'''**	24.7	24.7	0
**4'''**	25.0	25.0	0
**5'''**	46.9	46.9	0

[a]Measured at 400 MHz; [b]Measured at 500 MHz.

4. NMR Spectra

LXE_L1_15

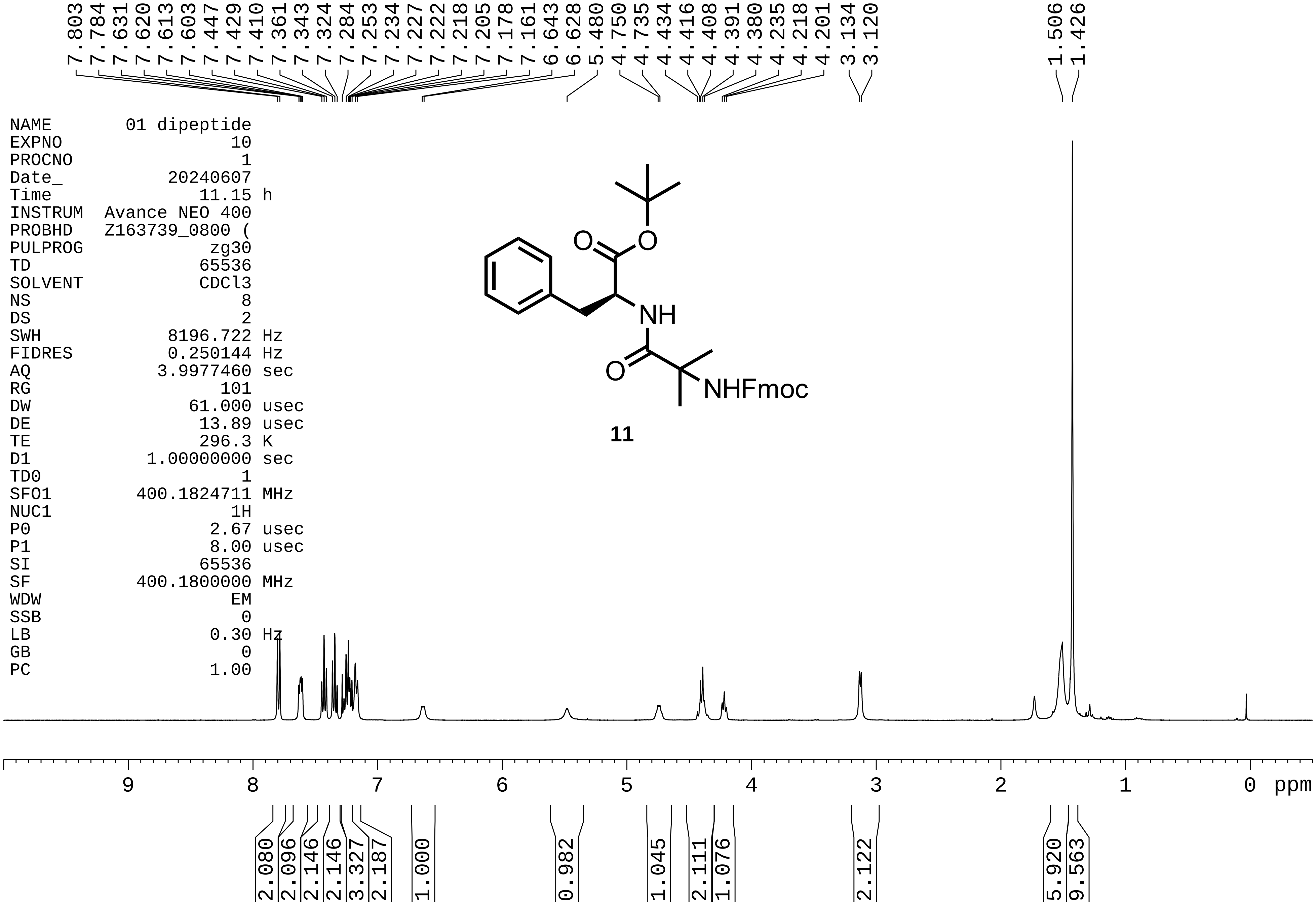

11

LXE_L1_15

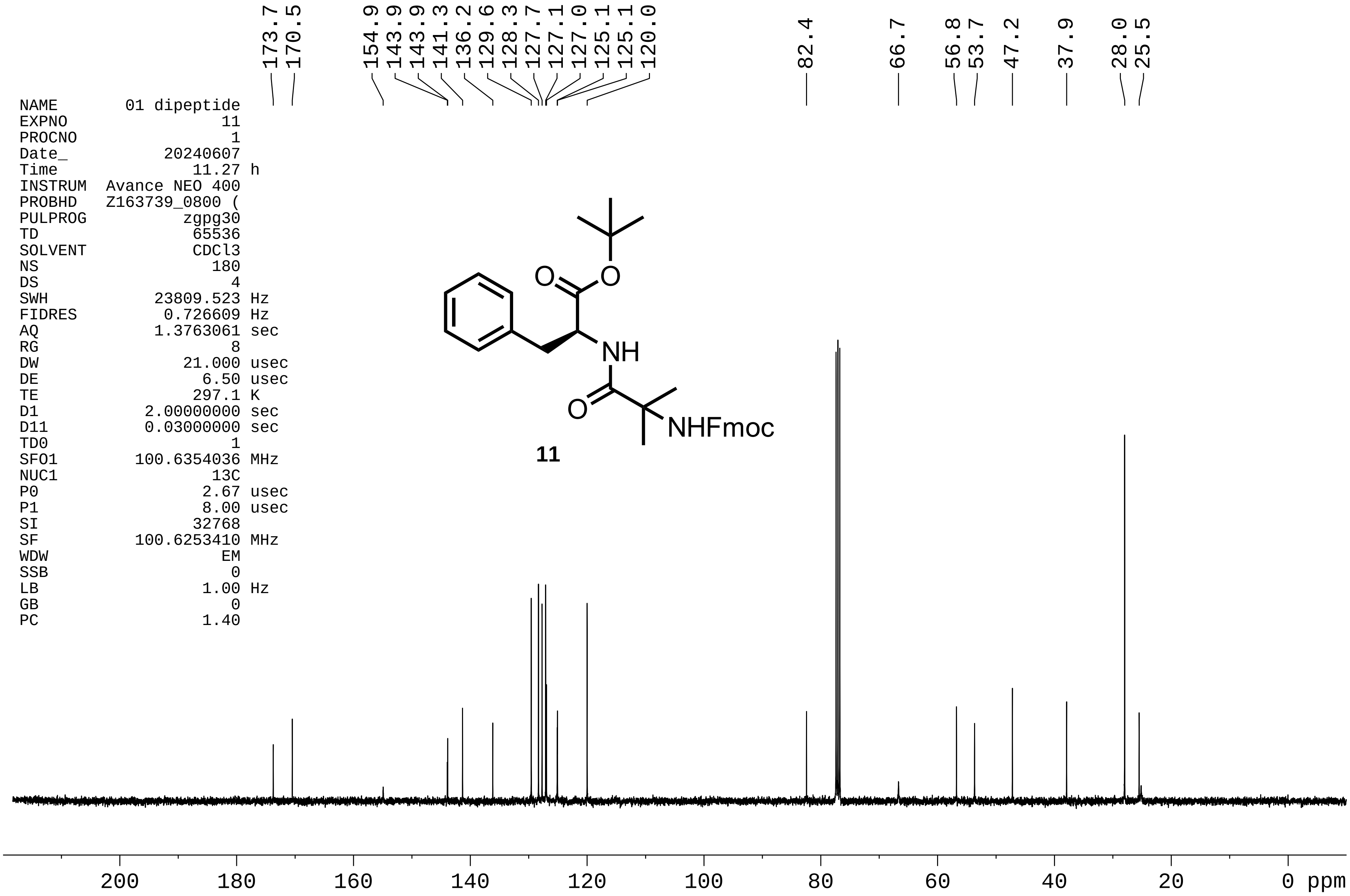
173.7
170.5
154.9
143.9
143.9
141.3
136.2
129.6
128.3
127.7
127.1
127.0
125.1
125.1
120.0
82.4
66.7
56.8
53.7
47.2
37.9
28.0
25.5
NAME 01 dipeptide
EXPNO 11
PROCNO 1
Date_ 20240607
Time 11.27 h
INSTRUM Avance NEO 400
PROBHD Z163739_0800 (
PULPROG zgpg30
TD 65536
SOLVENT CDCl3
NS 180
DS 4
SWH 23809.523 Hz
FIDRES 0.726609 Hz
AQ 1.3763061 sec
RG 8
DW 21.000 usec
DE 6.50 usec
TE 297.1 K
D1 2.00000000 sec
D11 0.03000000 sec
TD0 1
SFO1 100.6354036 MHz
NUC1 13C
P0 2.67 usec
P1 8.00 usec
SI 32768
SF 100.6253410 MHz
WDW EM
SSB 0
LB 1.00 Hz
GB 0
PC 1.40
O
O
NH
O
NHFmoc
11
200
180
160
140
120
100
80
60
40
20
0 ppm

LXE_KB_F3

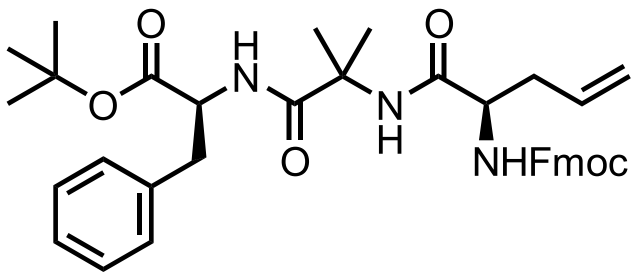
O
H
N
O
N
H
O
NHFmoc

12

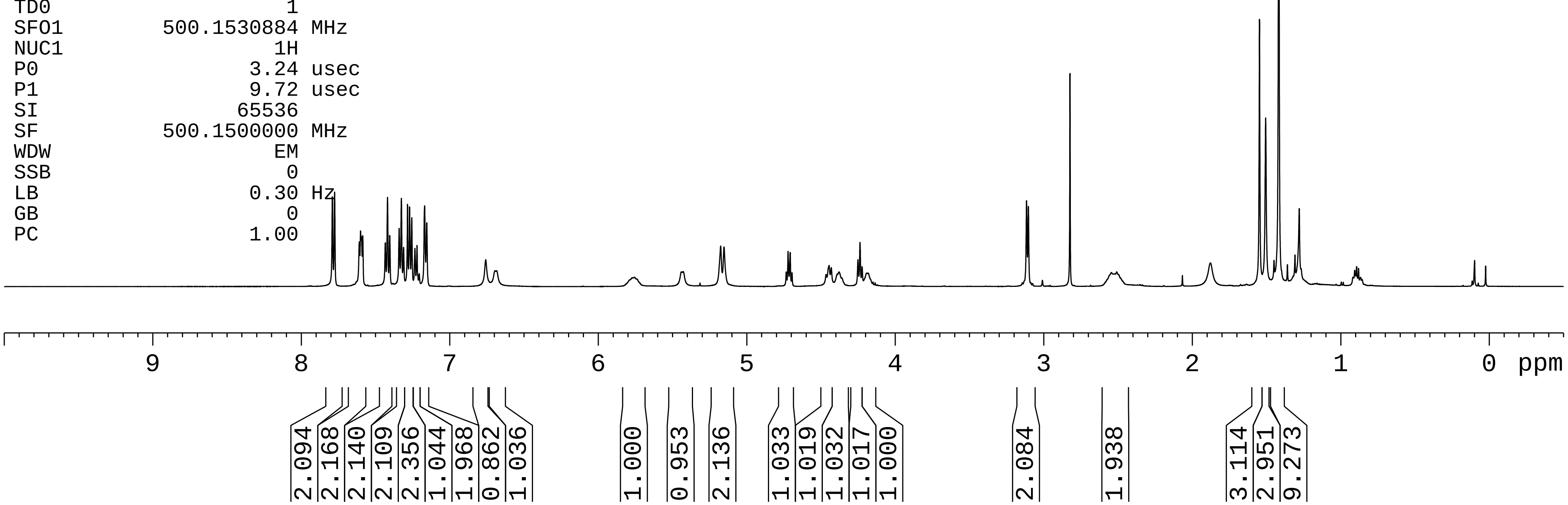
7.775
7.609
7.600
7.595
7.586
7.434
7.419
7.404
7.340
7.325
7.311
7.284
7.270
7.255
7.234
7.220
7.169
7.155
6.758
6.696
6.684
5.771
5.757
5.440
5.428
5.176
5.154
4.735
4.723
4.708
4.696
4.466
4.446
4.431
4.391
4.379
4.360
4.252
4.238
4.224
4.194
4.183
3.116
3.104
2.545
2.524
2.509
1.548
1.506
1.418
NAME 02 tripeptide
EXPNO 10
PROCNO 1
Date_ 20240810
Time 18.04 h
INSTRUM Avance NEO 500
PROBHD Z119470_0332 (
PULPROG zg30
TD 65536
SOLVENT CDCl3
NS 16
DS 2
SWH 10000.000 Hz
FIDRES 0.305176 Hz
AQ 3.2768500 sec
RG 101
DW 50.000 usec
DE 10.84 usec
TE 296.4 K
D1 1.00000000 sec
TD0 1
SFO1 500.1530884 MHz
NUC1 1H
P0 3.24 usec
P1 9.72 usec
SI 65536
SF 500.1500000 MHz
WDW EM
SSB 0
LB 0.30 Hz
GB 0
PC 1.00
9
8
7
6
5
4
3
2
1
0 ppm
2.094
2.168
2.140
2.109
2.356
1.044
1.968
0.862
1.036
1.000
0.953
2.136
1.033
1.019
1.032
1.017
1.000
2.084
1.938
3.114
2.951
9.273

LXE_KB_F3

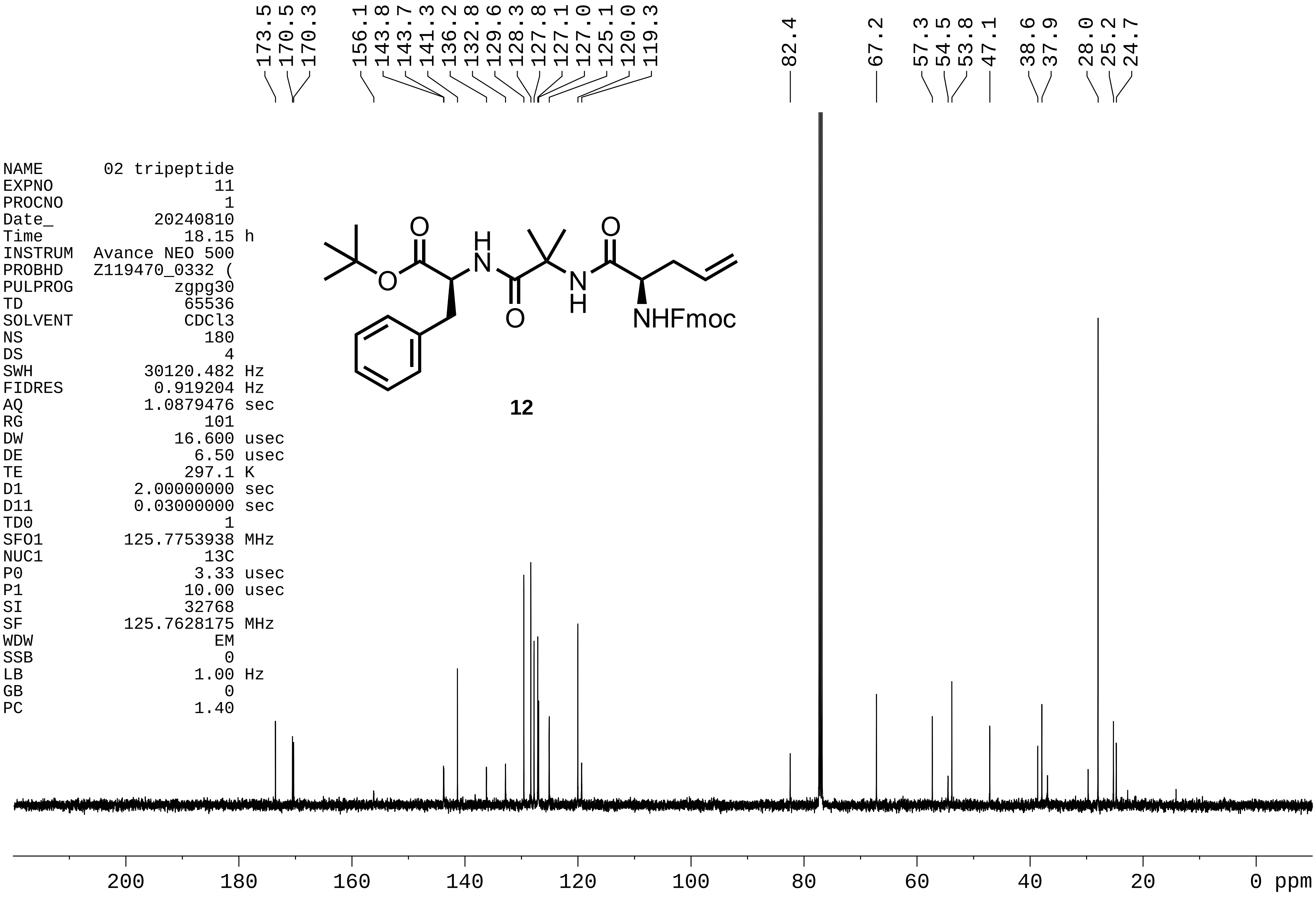

NAME	02 tripeptide	
EXPNO	11	
PROCNO	1	
Date_	20240810	
Time	18.15	h
INSTRUM	Avance NEO 500	
PROBHD	Z119470_0332 (	
PULPROG	zgpg30	
TD	65536	
SOLVENT	CDCl3	
NS	180	
DS	4	
SWH	30120.482	Hz
FIDRES	0.919204	Hz
AQ	1.0879476	sec
RG	101	
DW	16.600	usec
DE	6.50	usec
TE	297.1	K
D1	2.00000000	sec
D11	0.03000000	sec
TD0	1	
SFO1	125.7753938	MHz
NUC1	13C	
P0	3.33	usec
P1	10.00	usec
SI	32768	
SF	125.7628175	MHz
WDW	EM	
SSB	0	
LB	1.00	Hz
GB	0	
PC	1.40	

12

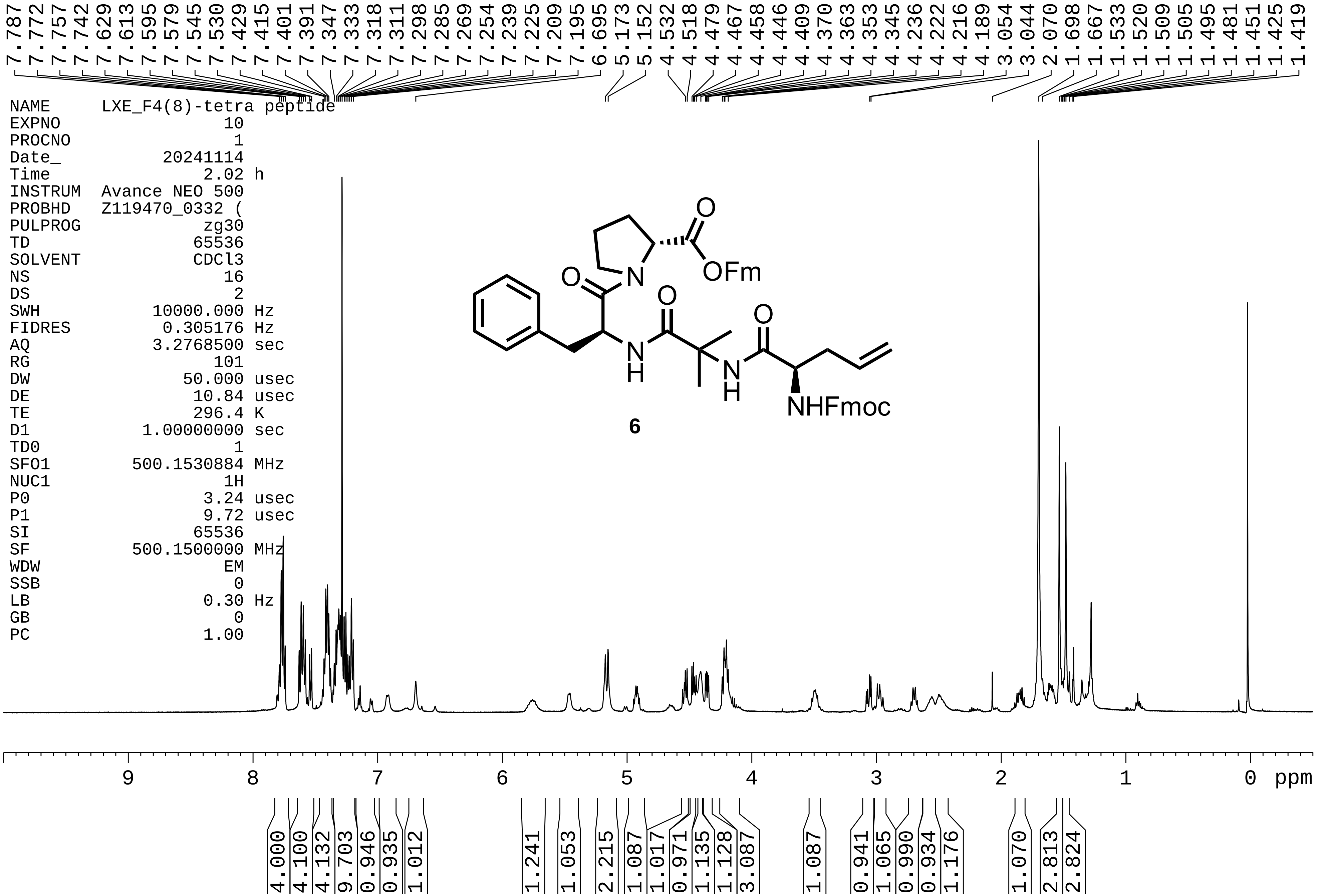
NAME LXE_F4(8)-tetra peptide
EXPNO 10
PROCNO 1
Date_ 20241114
Time 2.02 h
INSTRUM Avance NEO 500
PROBHD Z119470_0332 (
PULPROG zg30
TD 65536
SOLVENT CDCl3
NS 16
DS 2
SWH 10000.000 Hz
FIDRES 0.305176 Hz
AQ 3.2768500 sec
RG 101
DW 50.000 usec
DE 10.84 usec
TE 296.4 K
D1 1.00000000 sec
TD0 1
SFO1 500.1530884 MHz
NUC1 1H
P0 3.24 usec
P1 9.72 usec
SI 65536
SF 500.1500000 MHz
WDW EM
SSB 0
LB 0.30 Hz
GB 0
PC 1.00
7.787 7.772 7.757 7.742 7.629 7.613 7.595 7.579 7.545 7.530 7.429 7.415 7.401 7.391 7.347 7.333 7.318 7.311 7.298 7.285 7.269 7.254 7.239 7.225 7.209 7.195 6.695 5.173 5.152 4.532 4.518 4.479 4.467 4.458 4.446 4.409 4.370 4.363 4.353 4.345 4.236 4.222 4.216 4.189 3.054 3.044 2.070 1.698 1.667 1.533 1.520 1.509 1.505 1.495 1.481 1.451 1.425 1.419
O
OFm
O
N
O
O
N
H
N
H
NHFmoc
6
9 8 7 6 5 4 3 2 1 0 ppm
4.000 4.100 4.132 9.703 0.946 0.935 1.012 1.241 1.053 2.215 1.087 1.017 0.971 1.135 1.128 3.087 1.087 0.941 1.065 0.990 0.934 1.176 1.070 2.813 2.824

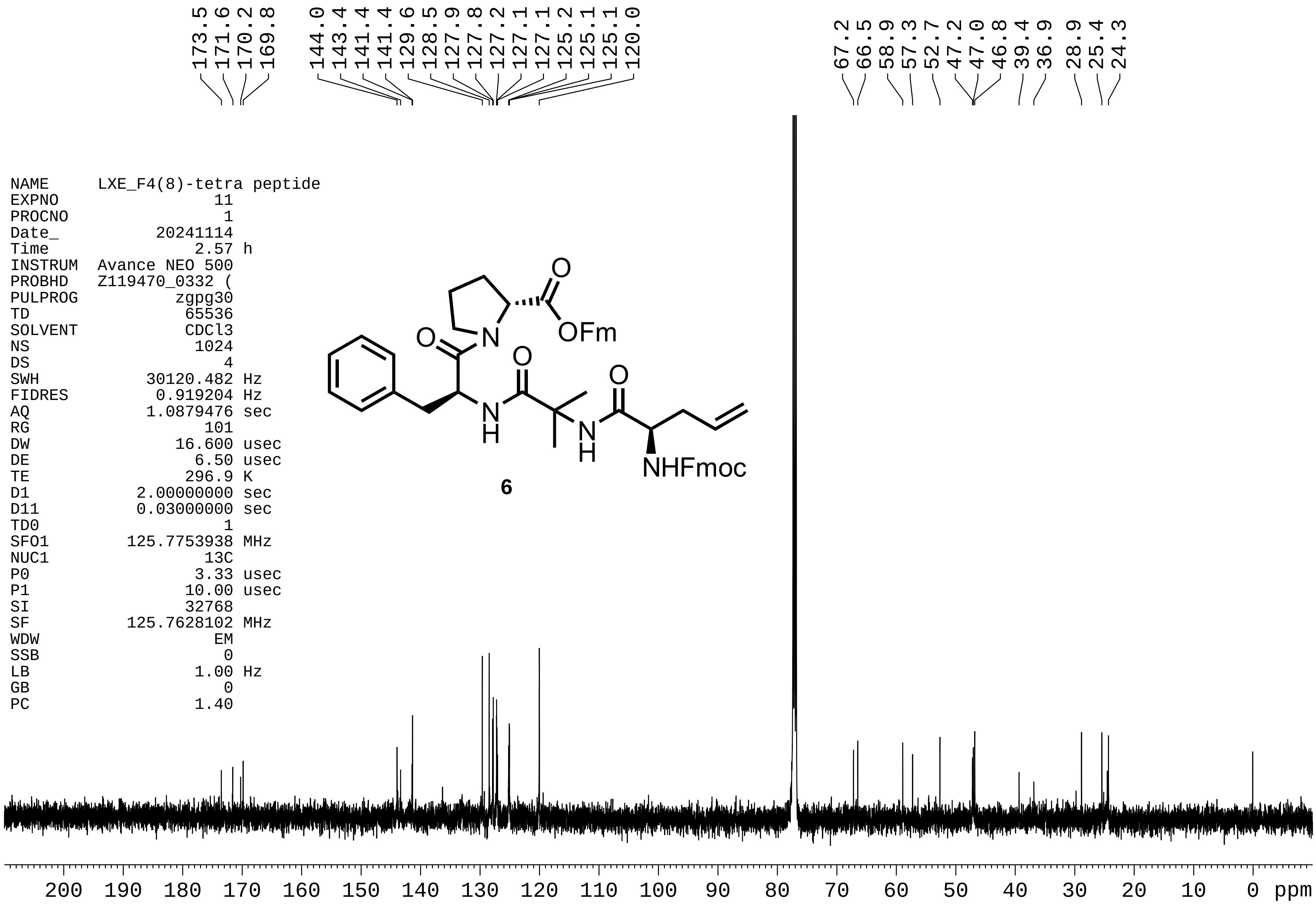
173.5
171.6
170.2
169.8
144.0
143.4
141.4
141.4
129.6
128.5
127.9
127.8
127.2
127.1
127.1
125.2
125.1
125.1
120.0
67.2
66.5
58.9
57.3
52.7
47.2
47.0
46.8
39.4
36.9
28.9
25.4
24.3
NAME LXE_F4(8)-tetra peptide
EXPNO 11
PROCNO 1
Date_ 20241114
Time 2.57 h
INSTRUM Avance NEO 500
PROBHD Z119470_0332 (
PULPROG zgpg30
TD 65536
SOLVENT CDCl3
NS 1024
DS 4
SWH 30120.482 Hz
FIDRES 0.919204 Hz
AQ 1.0879476 sec
RG 101
DW 16.600 usec
DE 6.50 usec
TE 296.9 K
D1 2.00000000 sec
D11 0.03000000 sec
TD0 1
SFO1 125.7753938 MHz
NUC1 13C
P0 3.33 usec
P1 10.00 usec
SI 32768
SF 125.7628102 MHz
WDW EM
SSB 0
LB 1.00 Hz
GB 0
PC 1.40
O
OFm
N
O
O
O
N
H
N
H
NHFmoc
200 190 180 170 160 150 140 130 120 110 100 90 80 70 60 50 40 30 20 10 0 ppm

6

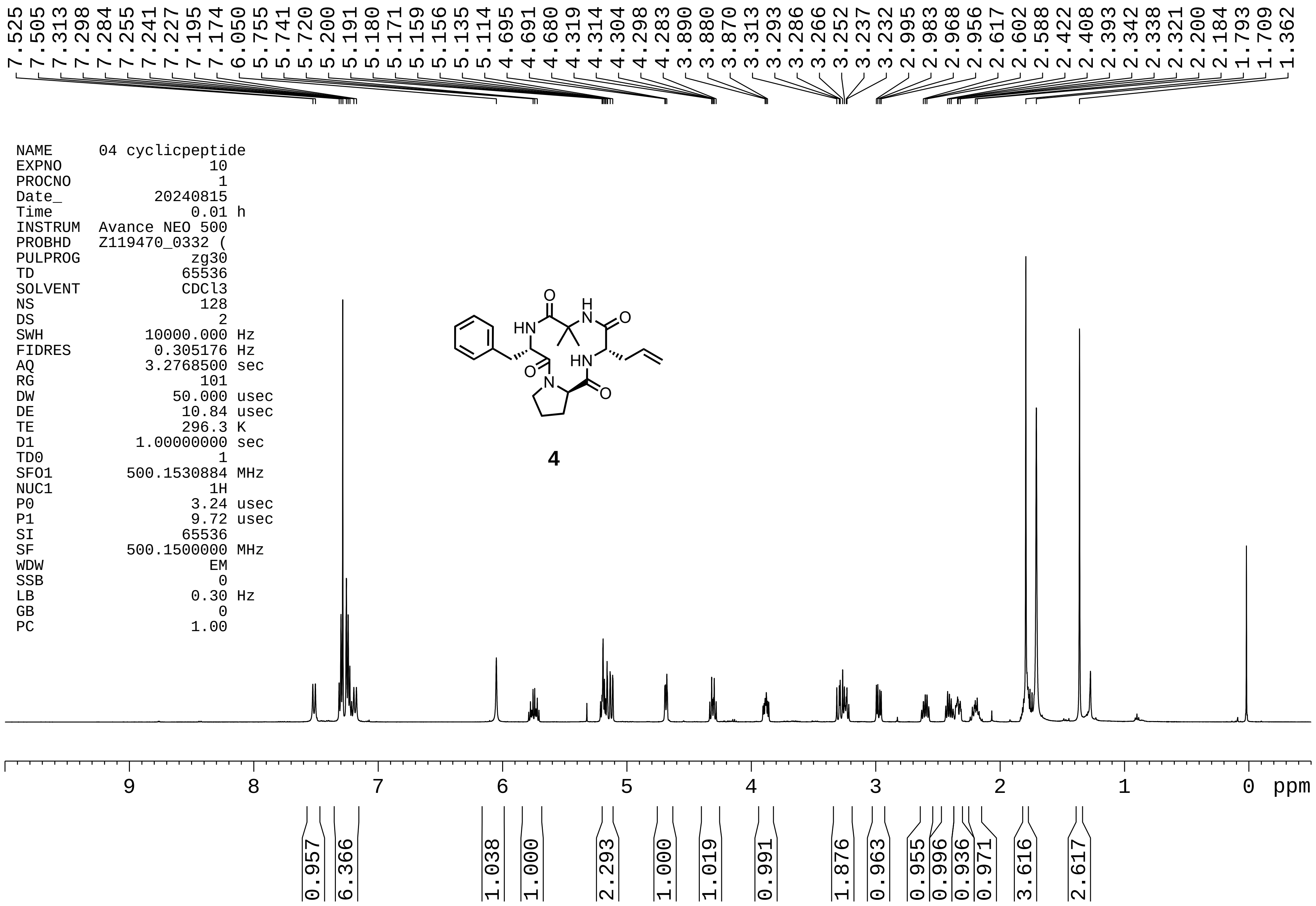
7.525 7.505 7.313 7.298 7.284 7.255 7.241 7.227 7.195 7.174 6.050 5.755 5.741 5.720 5.200 5.191 5.180 5.171 5.159 5.156 5.135 5.114 4.695 4.691 4.680 4.319 4.314 4.304 4.298 4.283 3.890 3.880 3.870 3.313 3.293 3.286 3.266 3.252 3.237 3.232 2.995 2.983 2.968 2.956 2.617 2.602 2.588 2.422 2.408 2.393 2.342 2.338 2.321 2.200 2.184 1.793 1.709 1.362
NAME 04 cyclicpeptide
EXPNO 10
PROCNO 1
Date_ 20240815
Time 0.01 h
INSTRUM Avance NEO 500
PROBHD Z119470_0332 (
PULPROG zg30
TD 65536
SOLVENT CDCl3
NS 128
DS 2
SWH 10000.000 Hz
FIDRES 0.305176 Hz
AQ 3.2768500 sec
RG 101
DW 50.000 usec
DE 10.84 usec
TE 296.3 K
D1 1.00000000 sec
TD0 1
SFO1 500.1530884 MHz
NUC1 1H
P0 3.24 usec
P1 9.72 usec
SI 65536
SF 500.1500000 MHz
WDW EM
SSB 0
LB 0.30 Hz
GB 0
PC 1.00
4
9 8 7 6 5 4 3 2 1 0 ppm
0.957 6.366 1.038 1.000 2.293 1.000 1.019 0.991 1.876 0.963 0.955 0.996 0.936 0.971 3.616 2.617

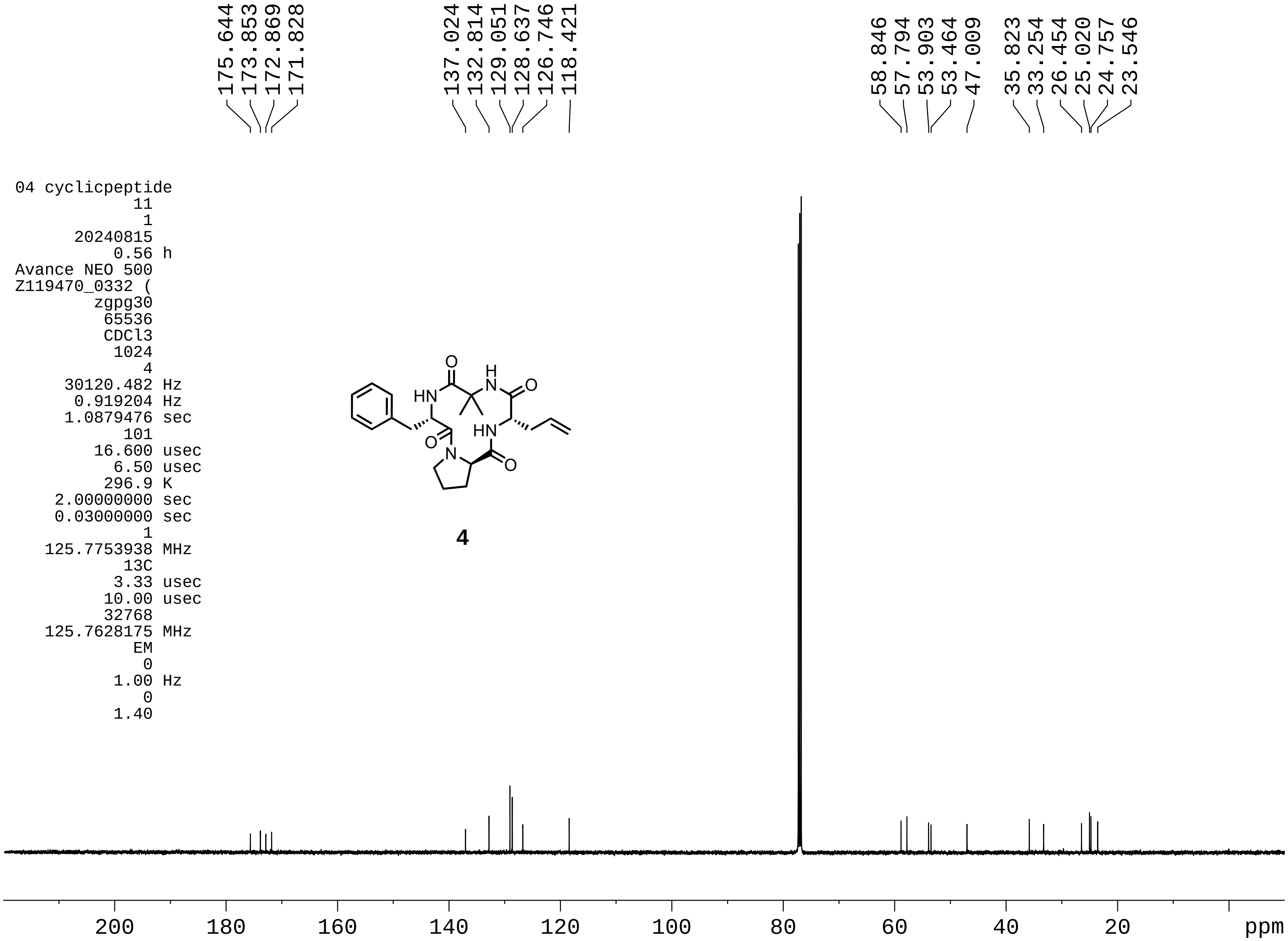

NAME	04 cyclicpeptide	
EXPNO	11	
PROCNO	1	
Date_	20240815	
Time	0.56	h
INSTRUM	Avance NEO 500	
PROBHD	Z119470_0332 (	
PULPROG	zgpg30	
TD	65536	
SOLVENT	CDCl3	
NS	1024	
DS	4	
SWH	30120.482	Hz
FIDRES	0.919204	Hz
AQ	1.0879476	sec
RG	101	
DW	16.600	usec
DE	6.50	usec
TE	296.9	K
D1	2.00000000	sec
D11	0.03000000	sec
TD0	1	
SFO1	125.7753938	MHz
NUC1	13C	
P0	3.33	usec
P1	10.00	usec
SI	32768	
SF	125.7628175	MHz
WDW	EM	
SSB	0	
LB	1.00	Hz
GB	0	
PC	1.40	

4

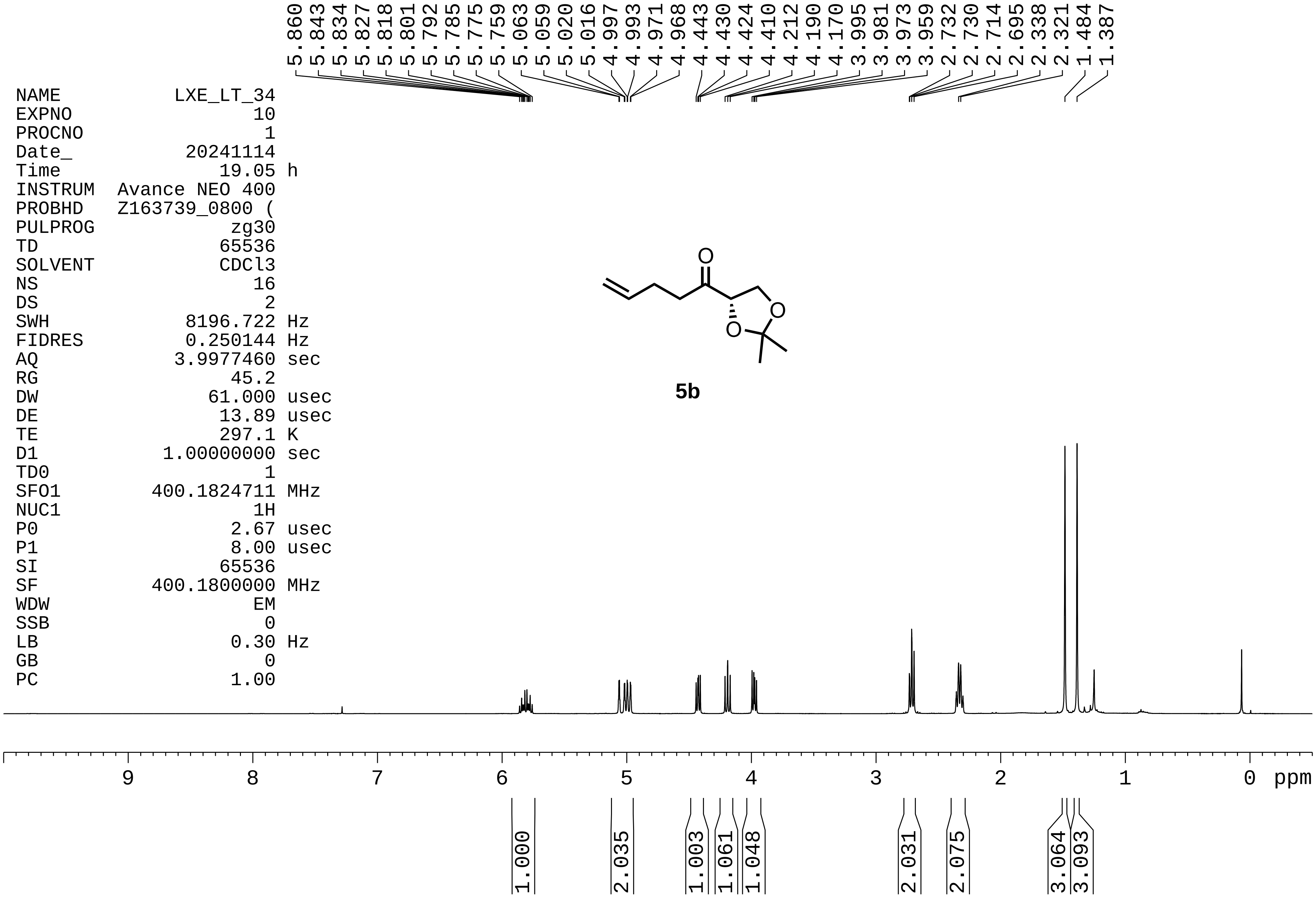
NAME LXE_LT_34
EXPNO 10
PROCNO 1
Date_ 20241114
Time 19.05 h
INSTRUM Avance NEO 400
PROBHD Z163739_0800 (
PULPROG zg30
TD 65536
SOLVENT CDCl3
NS 16
DS 2
SWH 8196.722 Hz
FIDRES 0.250144 Hz
AQ 3.9977460 sec
RG 45.2
DW 61.000 usec
DE 13.89 usec
TE 297.1 K
D1 1.00000000 sec
TD0 1
SFO1 400.1824711 MHz
NUC1 1H
P0 2.67 usec
P1 8.00 usec
SI 65536
SF 400.1800000 MHz
WDW EM
SSB 0
LB 0.30 Hz
GB 0
PC 1.00
5.860 5.843 5.834 5.827 5.818 5.801 5.792 5.785 5.775 5.759 5.063 5.059 5.020 5.016 4.997 4.993 4.971 4.968 4.443 4.430 4.424 4.410 4.212 4.190 4.170 3.995 3.981 3.973 3.959 2.732 2.730 2.714 2.695 2.338 2.321 1.484 1.387
5b
9 8 7 6 5 4 3 2 1 0 ppm
1.000 2.035 1.003 1.061 1.048 2.031 2.075 3.064 3.093

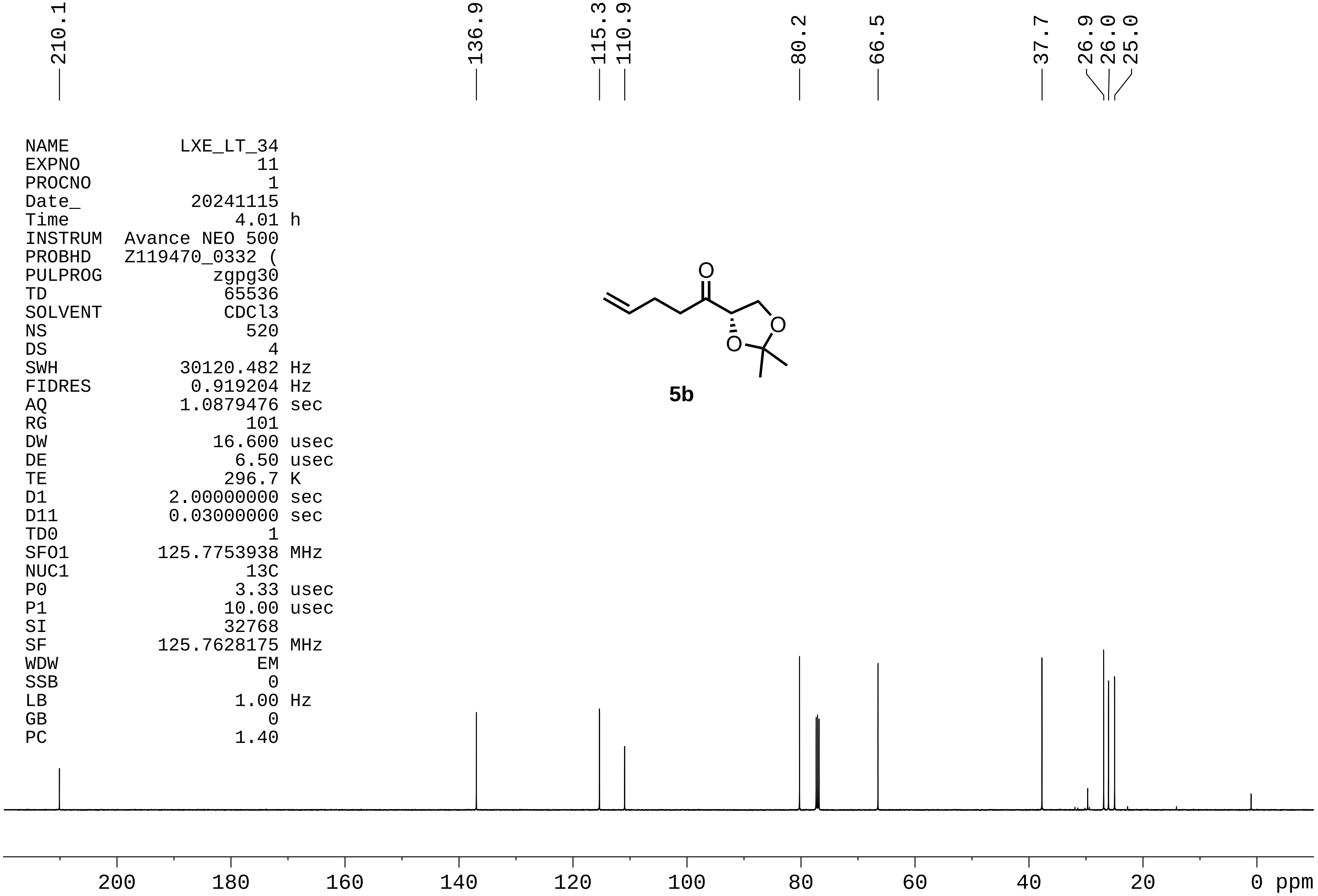

NAME	LXE_LT_34	
EXPNO	11	
PROCNO	1	
Date_	20241115	
Time	4.01	h
INSTRUM	Avance NEO 500	
PROBHD	Z119470_0332 (	
PULPROG	zgpg30	
TD	65536	
SOLVENT	CDCl3	
NS	520	
DS	4	
SWH	30120.482	Hz
FIDRES	0.919204	Hz
AQ	1.0879476	sec
RG	101	
DW	16.600	usec
DE	6.50	usec
TE	296.7	K
D1	2.00000000	sec
D11	0.03000000	sec
TD0	1	
SFO1	125.7753938	MHz
NUC1	13C	
P0	3.33	usec
P1	10.00	usec
SI	32768	
SF	125.7628175	MHz
WDW	EM	
SSB	0	
LB	1.00	Hz
GB	0	
PC	1.40	

5b

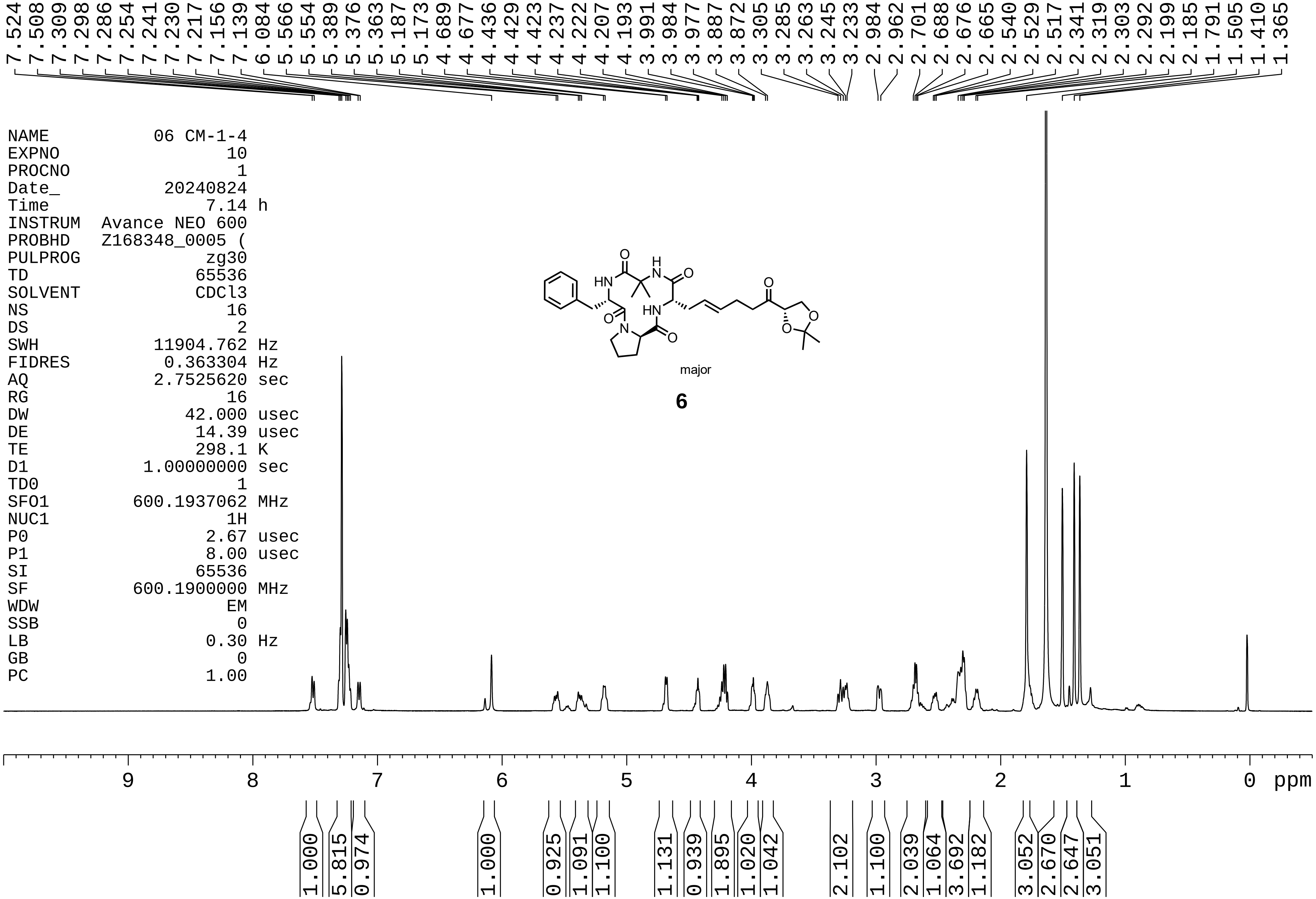
7.524 7.508 7.309 7.298 7.286 7.254 7.241 7.230 7.217 7.156 7.139 6.084 5.566 5.554 5.389 5.376 5.363 5.187 5.173 4.689 4.677 4.436 4.429 4.423 4.237 4.222 4.207 4.193 3.991 3.984 3.977 3.887 3.872 3.305 3.285 3.263 3.245 3.233 2.984 2.962 2.701 2.688 2.676 2.665 2.540 2.529 2.517 2.341 2.319 2.303 2.292 2.199 2.185 1.791 1.505 1.410 1.365
NAME 06 CM-1-4
EXPNO 10
PROCNO 1
Date_ 20240824
Time 7.14 h
INSTRUM Avance NEO 600
PROBHD Z168348_0005 (
PULPROG zg30
TD 65536
SOLVENT CDCl3
NS 16
DS 2
SWH 11904.762 Hz
FIDRES 0.363304 Hz
AQ 2.7525620 sec
RG 16
DW 42.000 usec
DE 14.39 usec
TE 298.1 K
D1 1.00000000 sec
TD0 1
SFO1 600.1937062 MHz
NUC1 1H
P0 2.67 usec
P1 8.00 usec
SI 65536
SF 600.1900000 MHz
WDW EM
SSB 0
LB 0.30 Hz
GB 0
PC 1.00
HN
O
H
N
O
O
HN
O
N
O
O
O
major
6
9 8 7 6 5 4 3 2 1 0 ppm
1.000 5.815 0.974 1.000 0.925 1.091 1.100 1.131 0.939 1.895 1.020 1.042 2.102 1.100 2.039 1.064 3.692 1.182 3.052 2.670 2.647 3.051

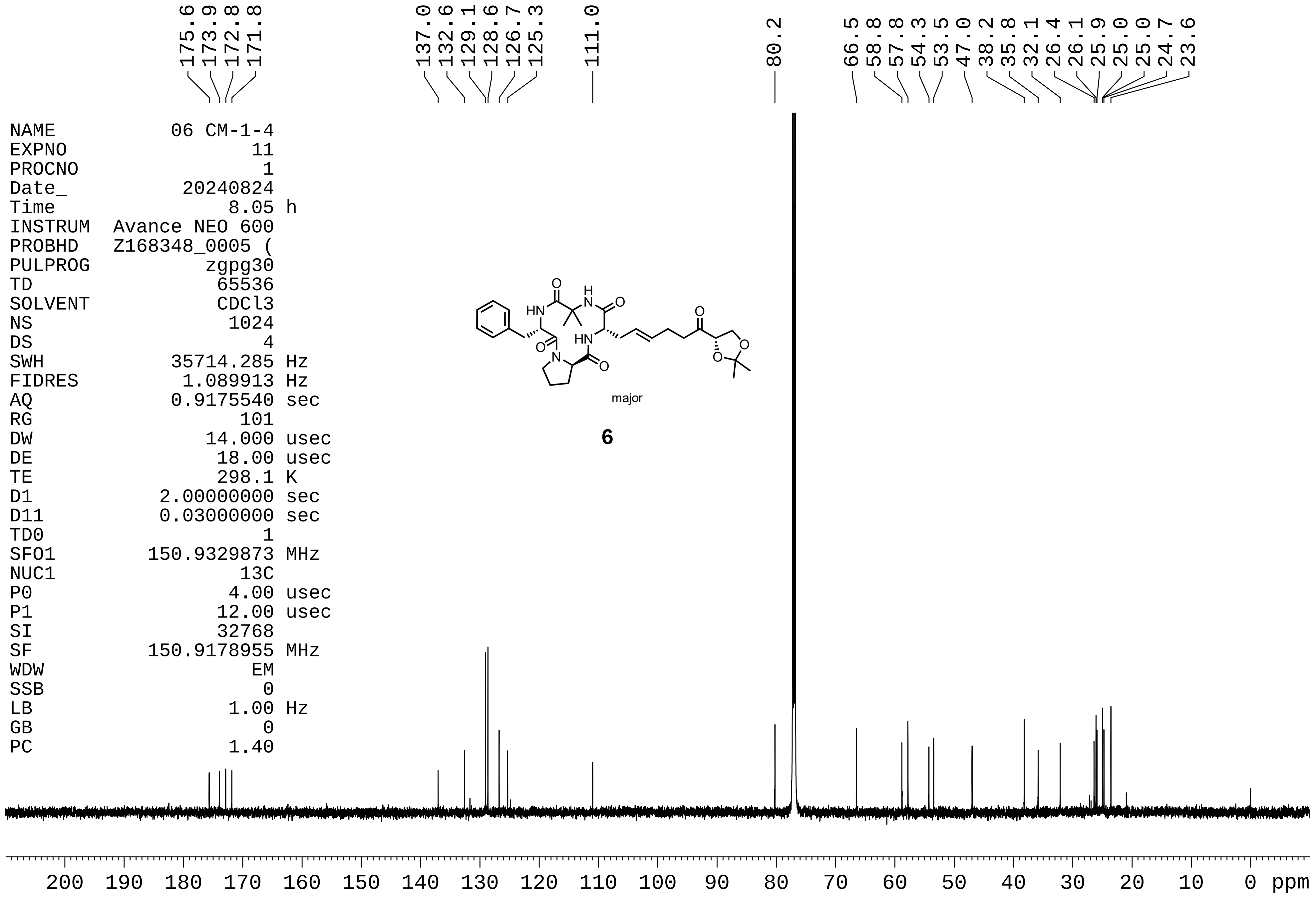
175.6
173.9
172.8
171.8
137.0
132.6
129.1
128.6
126.7
125.3
111.0
80.2
66.5
58.8
57.8
54.3
53.5
47.0
38.2
35.8
32.1
26.4
26.1
25.9
25.0
25.0
24.7
23.6
NAME 06 CM-1-4
EXPNO 11
PROCNO 1
Date_ 20240824
Time 8.05 h
INSTRUM Avance NEO 600
PROBHD Z168348_0005 (
PULPROG zgpg30
TD 65536
SOLVENT CDCl3
NS 1024
DS 4
SWH 35714.285 Hz
FIDRES 1.089913 Hz
AQ 0.9175540 sec
RG 101
DW 14.000 usec
DE 18.00 usec
TE 298.1 K
D1 2.00000000 sec
D11 0.03000000 sec
TD0 1
SFO1 150.9329873 MHz
NUC1 13C
P0 4.00 usec
P1 12.00 usec
SI 32768
SF 150.9178955 MHz
WDW EM
SSB 0
LB 1.00 Hz
GB 0
PC 1.40
major
6
200 190 180 170 160 150 140 130 120 110 100 90 80 70 60 50 40 30 20 10 0 ppm

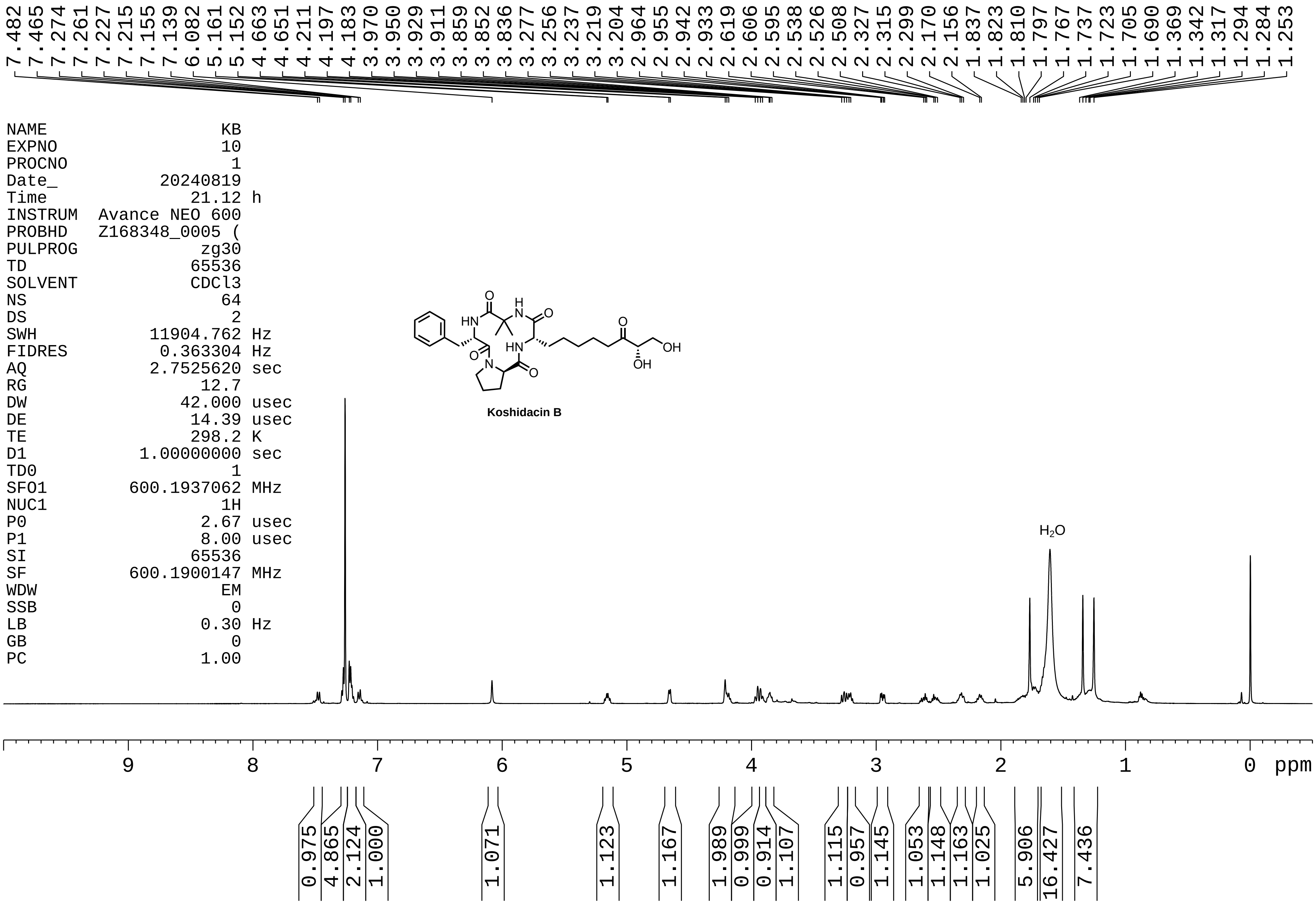
7.482 7.465 7.274 7.261 7.227 7.215 7.155 7.139 6.082 5.161 5.152 4.663 4.651 4.211 4.197 4.183 3.970 3.950 3.929 3.911 3.859 3.852 3.836 3.277 3.256 3.237 3.219 3.204 2.964 2.955 2.942 2.933 2.619 2.606 2.595 2.538 2.526 2.508 2.327 2.315 2.299 2.170 2.156 1.837 1.823 1.810 1.797 1.767 1.737 1.723 1.705 1.690 1.369 1.342 1.317 1.294 1.284 1.253
NAME KB
EXPNO 10
PROCNO 1
Date_ 20240819
Time 21.12 h
INSTRUM Avance NEO 600
PROBHD Z168348_0005 (
PULPROG zg30
TD 65536
SOLVENT CDCl3
NS 64
DS 2
SWH 11904.762 Hz
FIDRES 0.363304 Hz
AQ 2.7525620 sec
RG 12.7
DW 42.000 usec
DE 14.39 usec
TE 298.2 K
D1 1.00000000 sec
TD0 1
SFO1 600.1937062 MHz
NUC1 1H
P0 2.67 usec
P1 8.00 usec
SI 65536
SF 600.1900147 MHz
WDW EM
SSB 0
LB 0.30 Hz
GB 0
PC 1.00
Koshidacin B
H_2O
9 8 7 6 5 4 3 2 1 0 ppm
0.975 4.865 2.124 1.000 1.071 1.123 1.167 1.989 0.999 0.914 1.107 1.115 0.957 1.145 1.053 1.148 1.163 1.025 5.906 16.427 7.436

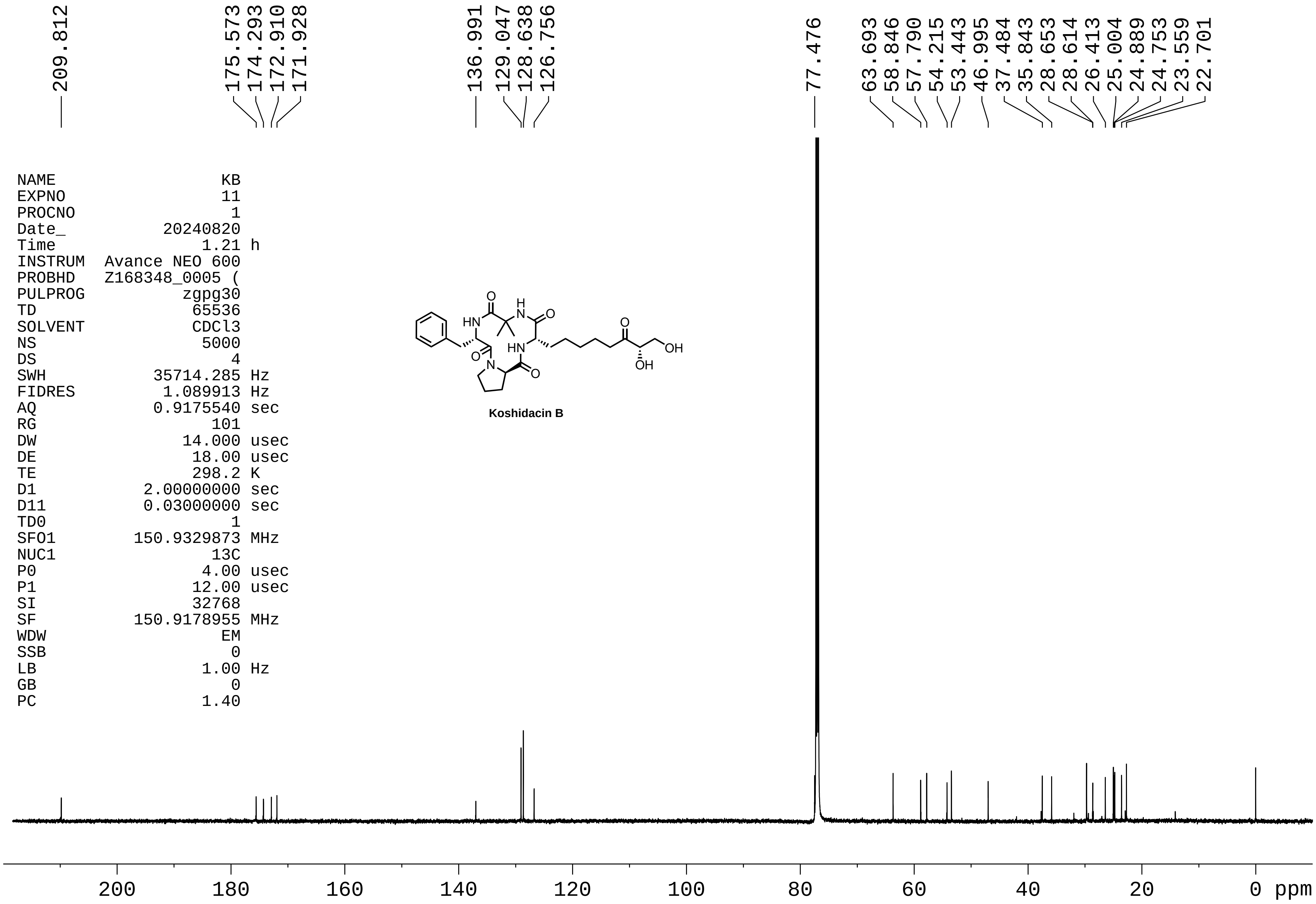
209.812
175.573
174.293
172.910
171.928
136.991
129.047
128.638
126.756
77.476
63.693
58.846
57.790
54.215
53.443
46.995
37.484
35.843
28.653
28.614
26.413
25.004
24.889
24.753
23.559
22.701
NAME KB
EXPNO 11
PROCNO 1
Date_ 20240820
Time 1.21 h
INSTRUM Avance NEO 600
PROBHD Z168348_0005 (
PULPROG zgpg30
TD 65536
SOLVENT CDCl3
NS 5000
DS 4
SWH 35714.285 Hz
FIDRES 1.089913 Hz
AQ 0.9175540 sec
RG 101
DW 14.000 usec
DE 18.00 usec
TE 298.2 K
D1 2.00000000 sec
D11 0.03000000 sec
TD0 1
SFO1 150.9329873 MHz
NUC1 13C
P0 4.00 usec
P1 12.00 usec
SI 32768
SF 150.9178955 MHz
WDW EM
SSB 0
LB 1.00 Hz
GB 0
PC 1.40
Koshidacin B
200
180
160
140
120
100
80
60
40
20
0 ppm

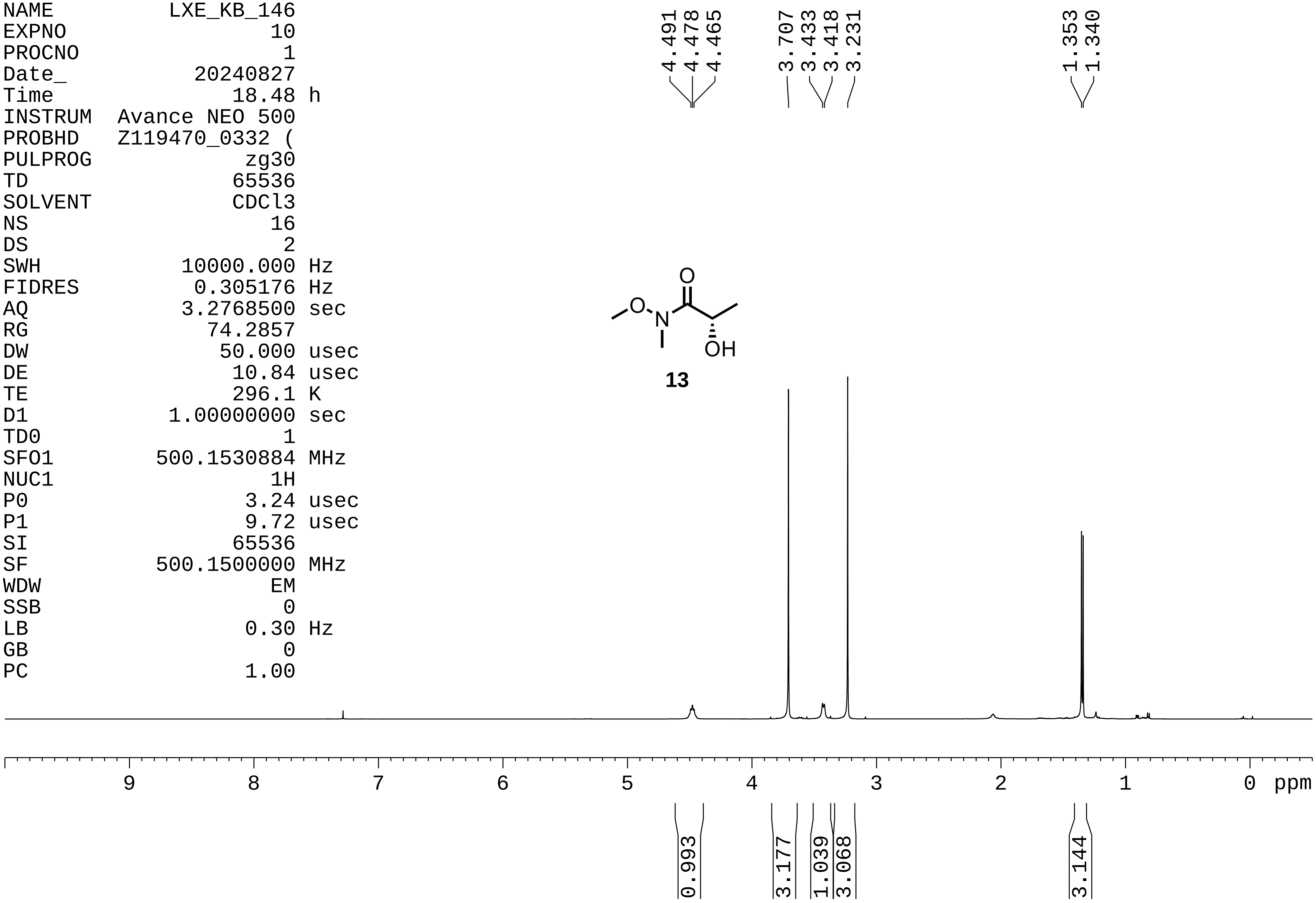
NAME LXE_KB_146
EXPNO 10
PROCNO 1
Date_ 20240827
Time 18.48 h
INSTRUM Avance NEO 500
PROBHD Z119470_0332 (
PULPROG zg30
TD 65536
SOLVENT CDCl3
NS 16
DS 2
SWH 10000.000 Hz
FIDRES 0.305176 Hz
AQ 3.2768500 sec
RG 74.2857
DW 50.000 usec
DE 10.84 usec
TE 296.1 K
D1 1.00000000 sec
TD0 1
SFO1 500.1530884 MHz
NUC1 1H
P0 3.24 usec
P1 9.72 usec
SI 65536
SF 500.1500000 MHz
WDW EM
SSB 0
LB 0.30 Hz
GB 0
PC 1.00
4.491
4.478
4.465
3.707
3.433
3.418
3.231
1.353
1.340
O
O
N
OH
13
9 8 7 6 5 4 3 2 1 0 ppm
0.993
3.177
1.039
3.068
3.144

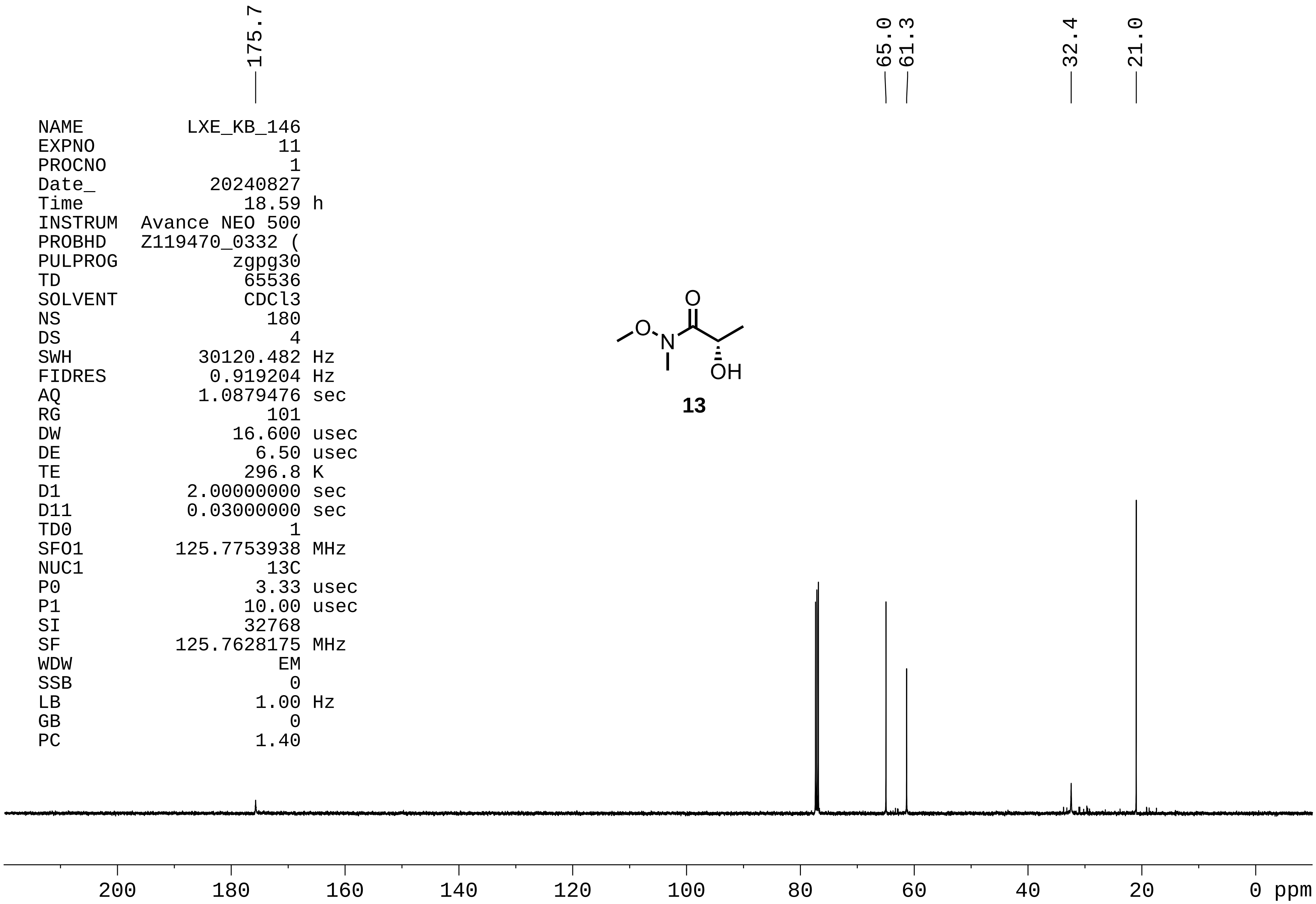
175.7
65.0
61.3
32.4
21.0
NAME LXE_KB_146
EXPNO 11
PROCNO 1
Date_ 20240827
Time 18.59 h
INSTRUM Avance NEO 500
PROBHD Z119470_0332 (
PULPROG zgpg30
TD 65536
SOLVENT CDCl3
NS 180
DS 4
SWH 30120.482 Hz
FIDRES 0.919204 Hz
AQ 1.0879476 sec
RG 101
DW 16.600 usec
DE 6.50 usec
TE 296.8 K
D1 2.00000000 sec
D11 0.03000000 sec
TD0 1
SFO1 125.7753938 MHz
NUC1 13C
P0 3.33 usec
P1 10.00 usec
SI 32768
SF 125.7628175 MHz
WDW EM
SSB 0
LB 1.00 Hz
GB 0
PC 1.40
O
O
N
OH
13
200
180
160
140
120
100
80
60
40
20
0 ppm

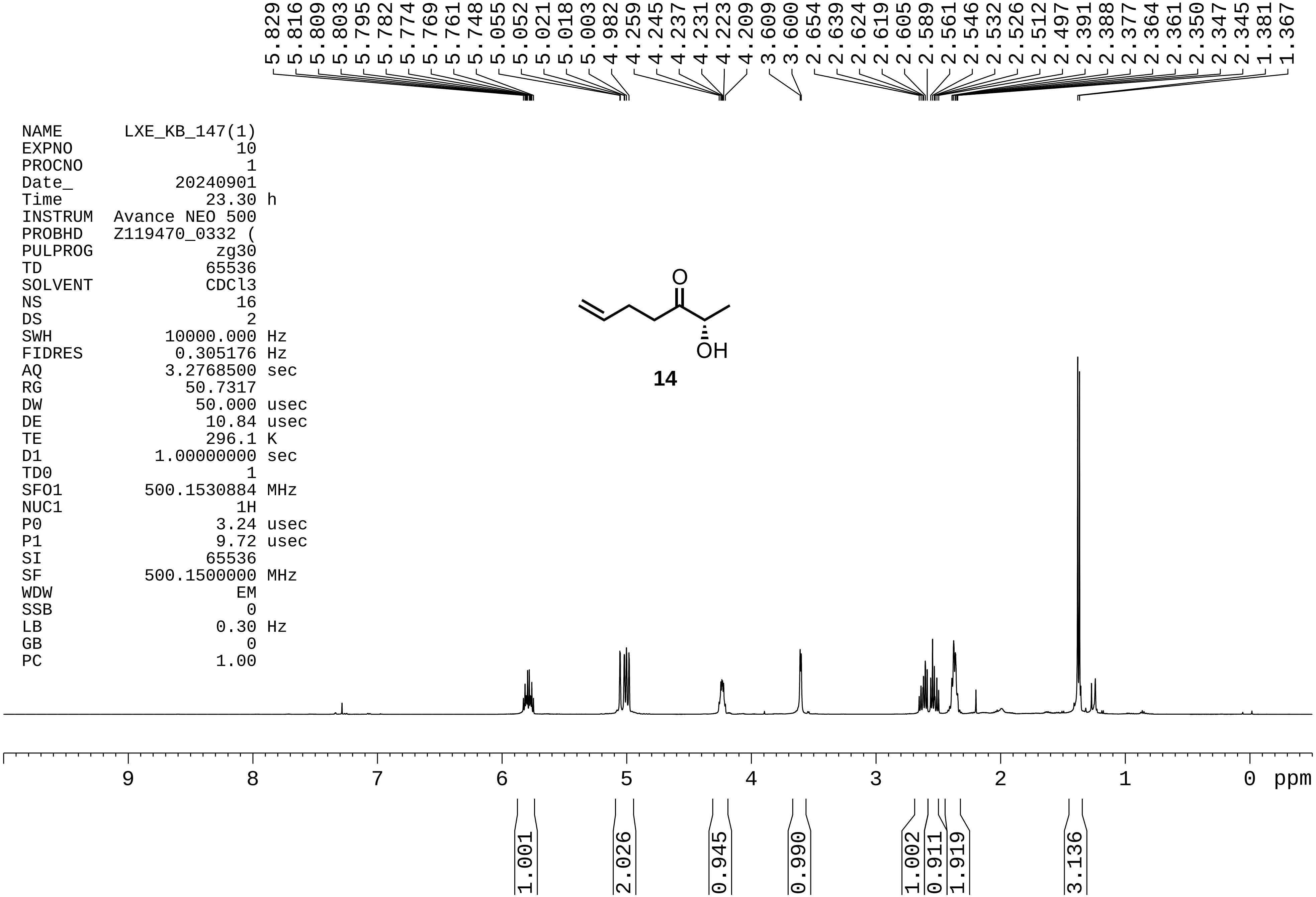
5.829 5.816 5.809 5.803 5.795 5.782 5.774 5.769 5.761 5.748 5.055 5.052 5.021 5.018 5.003 4.982 4.259 4.245 4.237 4.231 4.223 4.209 3.609 3.600 2.654 2.639 2.624 2.619 2.605 2.589 2.561 2.546 2.532 2.526 2.512 2.497 2.391 2.388 2.377 2.364 2.361 2.350 2.347 2.345 1.381 1.367
NAME LXE_KB_147(1)
EXPNO 10
PROCNO 1
Date_ 20240901
Time 23.30 h
INSTRUM Avance NEO 500
PROBHD Z119470_0332 (
PULPROG zg30
TD 65536
SOLVENT CDCl3
NS 16
DS 2
SWH 10000.000 Hz
FIDRES 0.305176 Hz
AQ 3.2768500 sec
RG 50.7317
DW 50.000 usec
DE 10.84 usec
TE 296.1 K
D1 1.00000000 sec
TD0 1
SFO1 500.1530884 MHz
NUC1 1H
P0 3.24 usec
P1 9.72 usec
SI 65536
SF 500.1500000 MHz
WDW EM
SSB 0
LB 0.30 Hz
GB 0
PC 1.00
O
OH
14
9
8
7
6
5
4
3
2
1
0 ppm
1.001
2.026
0.945
0.990
1.002
0.911
1.919
3.136

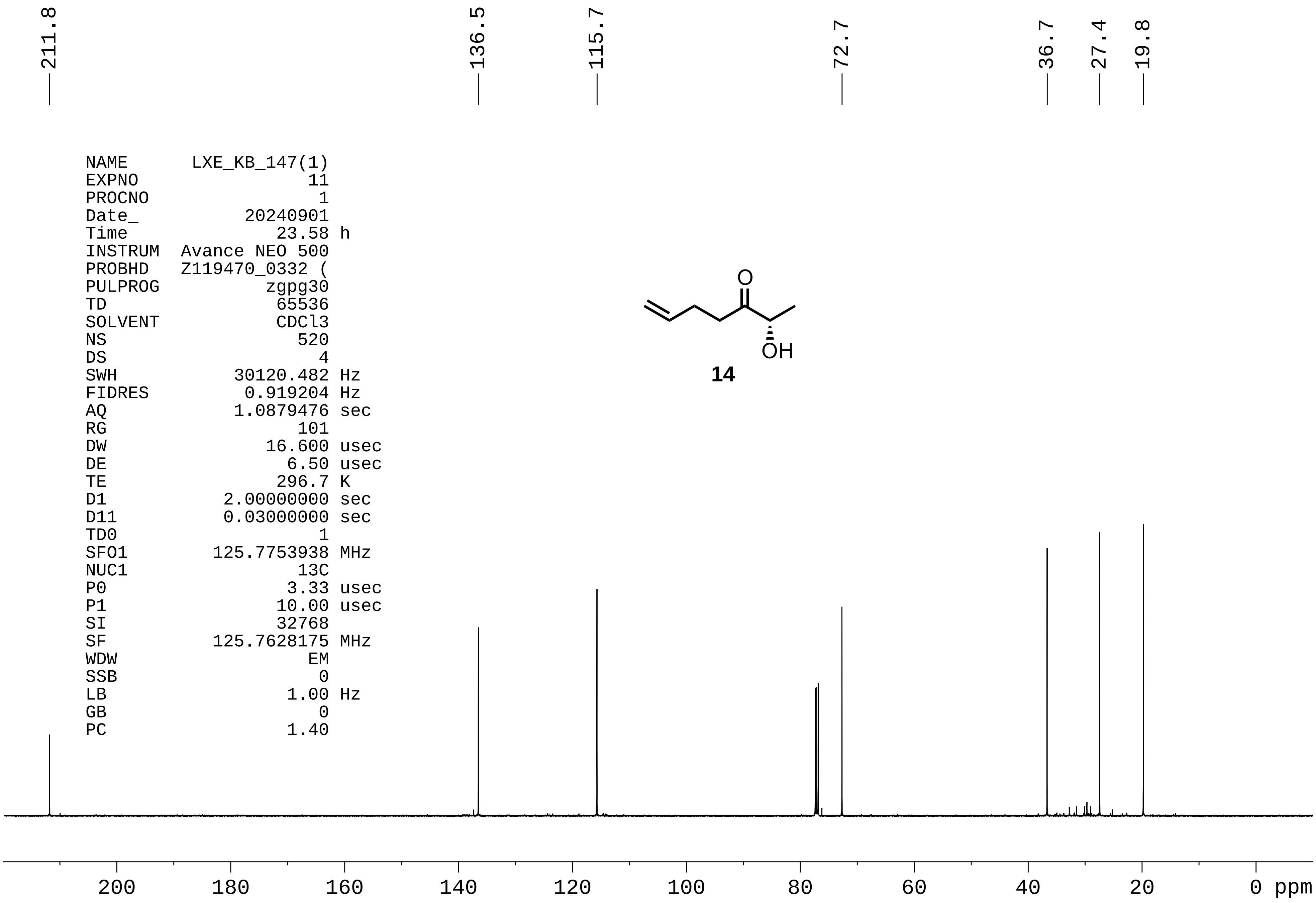
211.8
136.5
115.7
72.7
36.7
27.4
19.8
NAME LXE_KB_147(1)
EXPNO 11
PROCNO 1
Date_ 20240901
Time 23.58 h
INSTRUM Avance NEO 500
PROBHD Z119470_0332 (
PULPROG zgpg30
TD 65536
SOLVENT CDCl3
NS 520
DS 4
SWH 30120.482 Hz
FIDRES 0.919204 Hz
AQ 1.0879476 sec
RG 101
DW 16.600 usec
DE 6.50 usec
TE 296.7 K
D1 2.00000000 sec
D11 0.03000000 sec
TD0 1
SFO1 125.7753938 MHz
NUC1 13C
P0 3.33 usec
P1 10.00 usec
SI 32768
SF 125.7628175 MHz
WDW EM
SSB 0
LB 1.00 Hz
GB 0
PC 1.40
200
180
160
140
120
100
80
60
40
20
0 ppm
O
OH

14

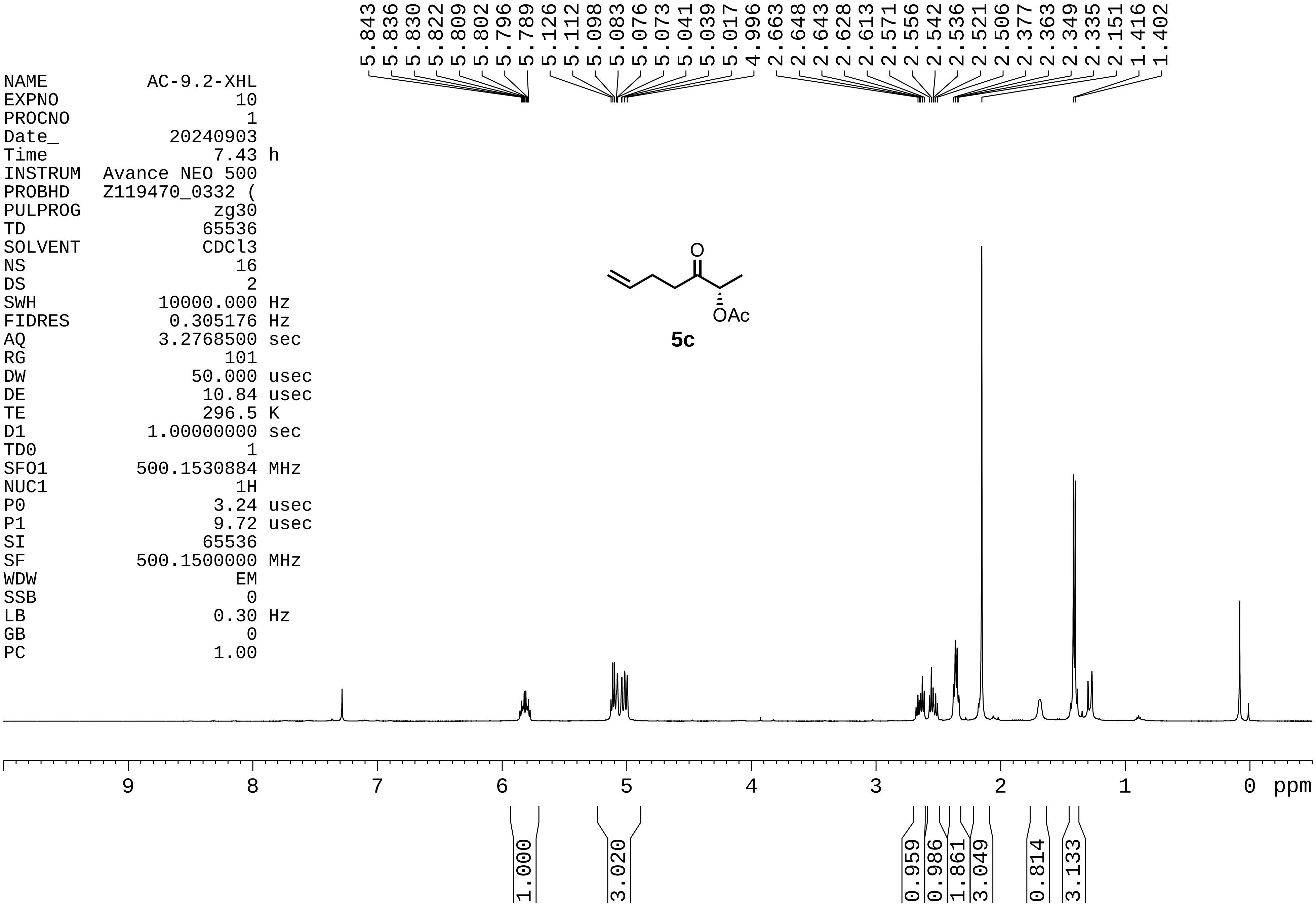
NAME AC-9.2-XHL
EXPNO 10
PROCNO 1
Date_ 20240903
Time 7.43 h
INSTRUM Avance NEO 500
PROBHD Z119470_0332 (
PULPROG zg30
TD 65536
SOLVENT CDCl3
NS 16
DS 2
SWH 10000.000 Hz
FIDRES 0.305176 Hz
AQ 3.2768500 sec
RG 101
DW 50.000 usec
DE 10.84 usec
TE 296.5 K
D1 1.00000000 sec
TD0 1
SFO1 500.1530884 MHz
NUC1 1H
P0 3.24 usec
P1 9.72 usec
SI 65536
SF 500.1500000 MHz
WDW EM
SSB 0
LB 0.30 Hz
GB 0
PC 1.00
5.843 5.836 5.830 5.822 5.809 5.802 5.796 5.789 5.126 5.112 5.098 5.083 5.076 5.073 5.041 5.039 5.017 4.996 2.663 2.648 2.643 2.628 2.613 2.571 2.556 2.542 2.536 2.521 2.506 2.377 2.363 2.349 2.335 2.151 1.416 1.402
O
OAc
5c
9 8 7 6 5 4 3 2 1 0 ppm
1.000 3.020 0.959 0.986 1.861 3.049 0.814 3.133

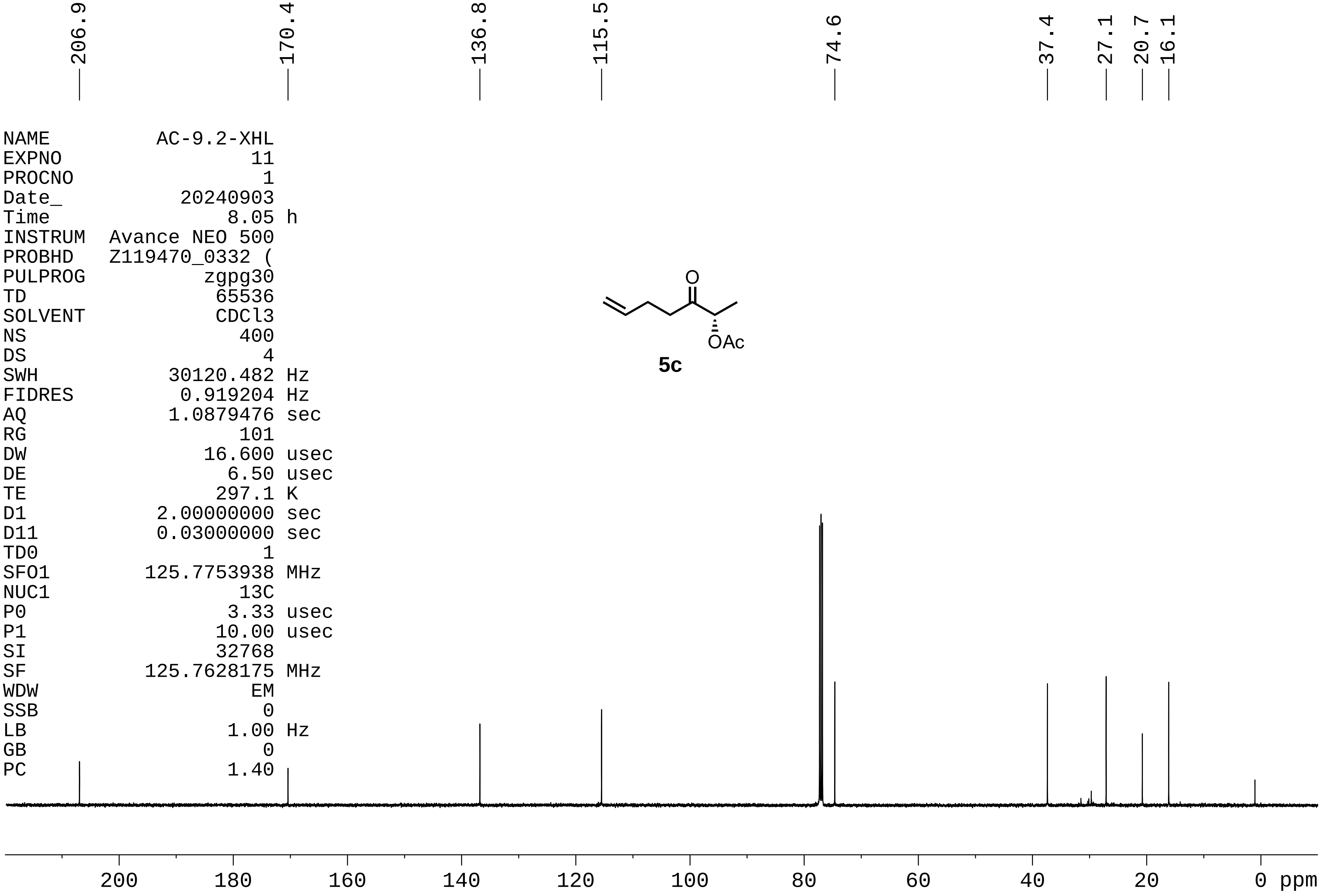
206.9
170.4
136.8
115.5
74.6
37.4
27.1
20.7
16.1
NAME AC-9.2-XHL
EXPNO 11
PROCNO 1
Date_ 20240903
Time 8.05 h
INSTRUM Avance NEO 500
PROBHD Z119470_0332 (
PULPROG zgpg30
TD 65536
SOLVENT CDCl3
NS 400
DS 4
SWH 30120.482 Hz
FIDRES 0.919204 Hz
AQ 1.0879476 sec
RG 101
DW 16.600 usec
DE 6.50 usec
TE 297.1 K
D1 2.00000000 sec
D11 0.03000000 sec
TD0 1
SFO1 125.7753938 MHz
NUC1 13C
P0 3.33 usec
P1 10.00 usec
SI 32768
SF 125.7628175 MHz
WDW EM
SSB 0
LB 1.00 Hz
GB 0
PC 1.40
O
OAc
5c
200
180
160
140
120
100
80
60
40
20
0 ppm

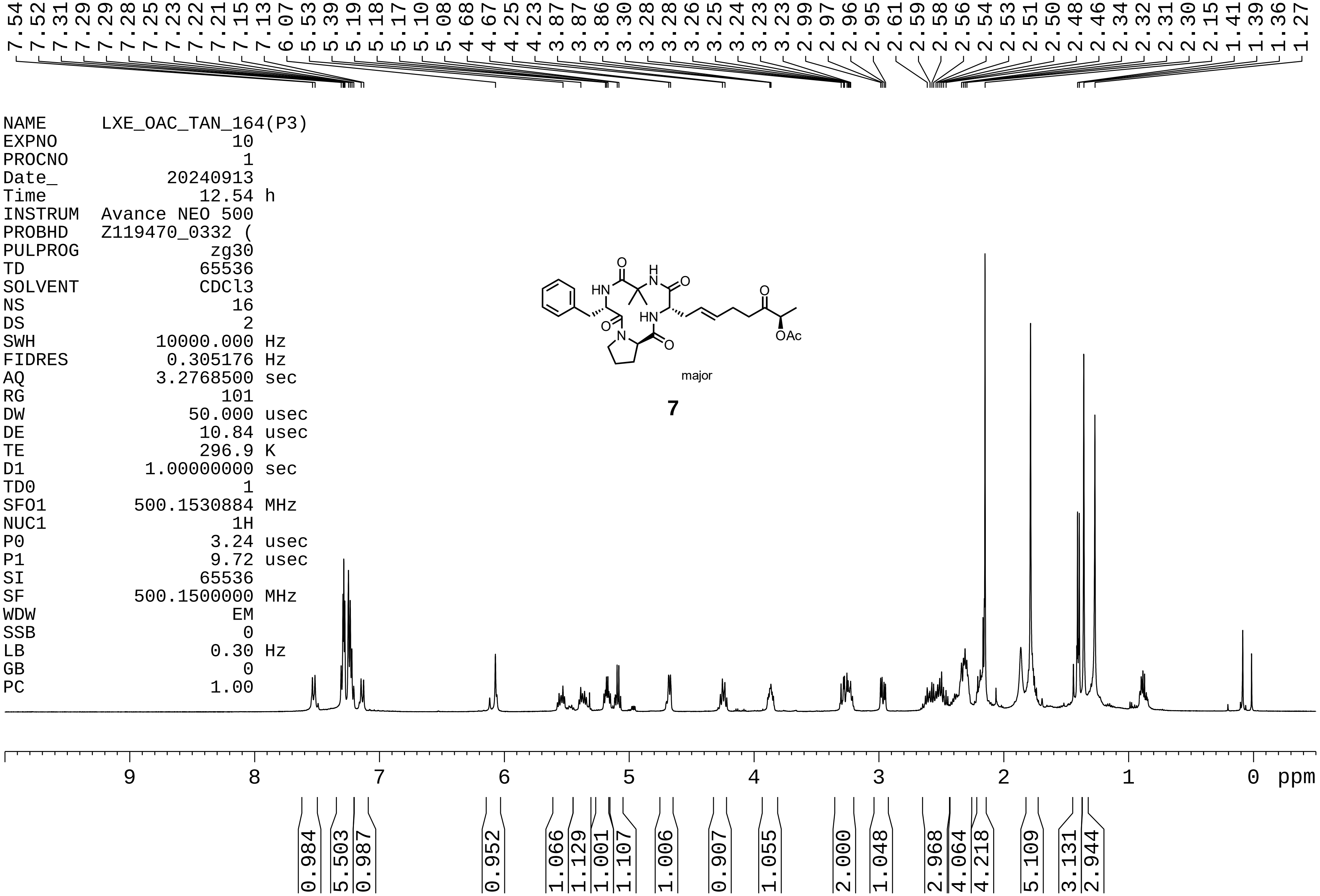

7.54 7.52 7.31 7.29 7.29 7.28 7.25 7.23 7.22 7.21 7.15 7.13 6.07 5.53 5.39 5.19 5.18 5.17 5.10 5.08 4.68 4.67 4.25 4.23 3.87 3.87 3.86 3.30 3.28 3.28 3.26 3.25 3.24 3.23 3.23 2.99 2.97 2.96 2.95 2.61 2.59 2.58 2.56 2.54 2.53 2.51 2.50 2.48 2.46 2.34 2.32 2.31 2.30 2.15 1.41 1.39 1.36 1.27
NAME LXE_OAC_TAN_164(P3)
EXPNO 10
PROCNO 1
Date_ 20240913
Time 12.54 h
INSTRUM Avance NEO 500
PROBHD Z119470_0332 (
PULPROG zg30
TD 65536
SOLVENT CDCl3
NS 16
DS 2
SWH 10000.000 Hz
FIDRES 0.305176 Hz
AQ 3.2768500 sec
RG 101
DW 50.000 usec
DE 10.84 usec
TE 296.9 K
D1 1.00000000 sec
TD0 1
SFO1 500.1530884 MHz
NUC1 1H
P0 3.24 usec
P1 9.72 usec
SI 65536
SF 500.1500000 MHz
WDW EM
SSB 0
LB 0.30 Hz
GB 0
PC 1.00
HN
H
N
O
OAc
major
7
9 8 7 6 5 4 3 2 1 0 ppm
0.984 5.503 0.987 0.952 1.066 1.129 1.001 1.107 1.006 0.907 1.055 2.000 1.048 2.968 4.064 4.218 5.109 3.131 2.944

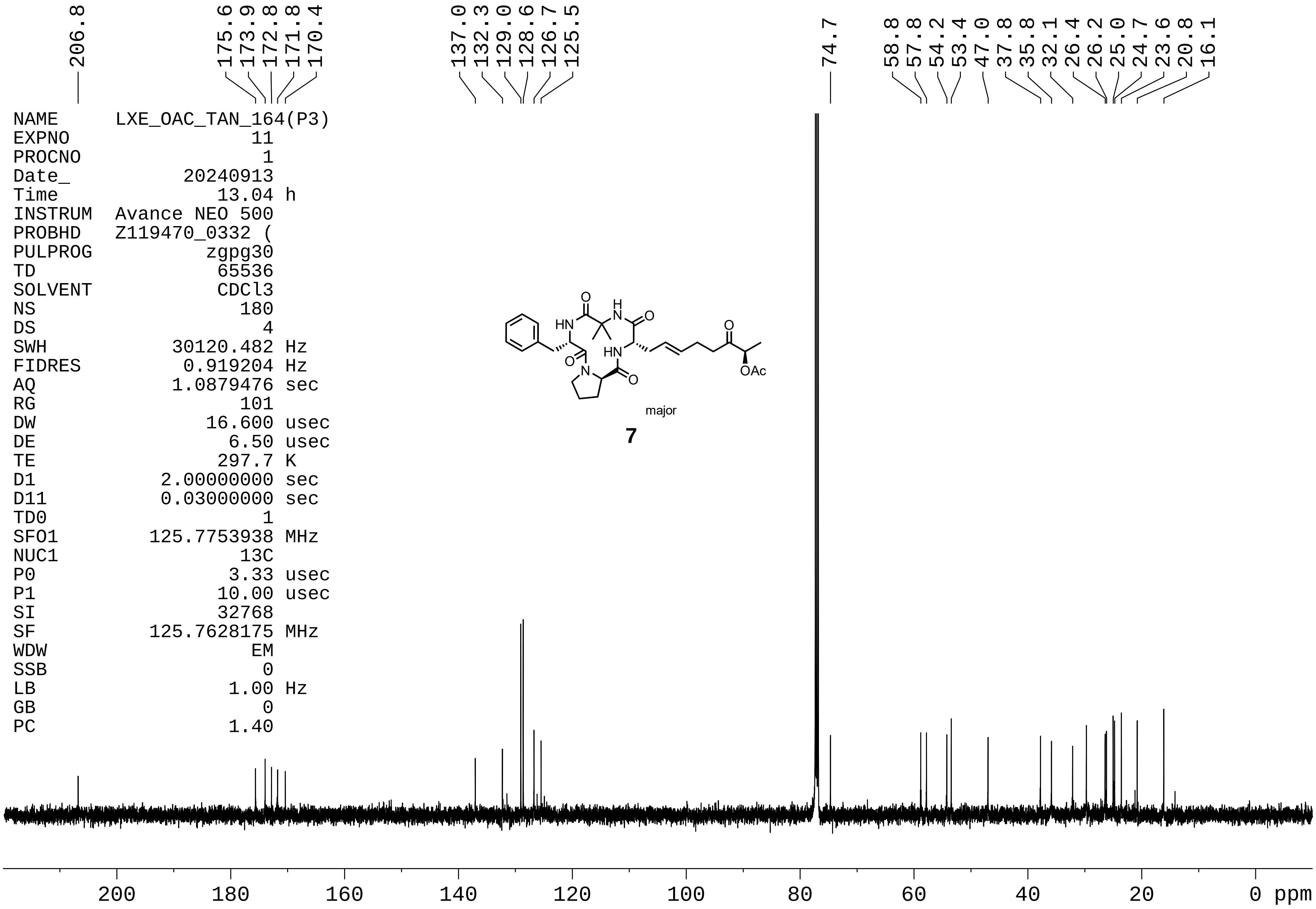
206.8
175.6
173.9
172.8
171.8
170.4
137.0
132.3
129.0
128.6
126.7
125.5
74.7
58.8
57.8
54.2
53.4
47.0
37.8
35.8
32.1
26.4
26.2
25.0
24.7
23.6
20.8
16.1
NAME LXE_OAC_TAN_164(P3)
EXPNO 11
PROCNO 1
Date_ 20240913
Time 13.04 h
INSTRUM Avance NEO 500
PROBHD Z119470_0332 (
PULPROG zgpg30
TD 65536
SOLVENT CDCl3
NS 180
DS 4
SWH 30120.482 Hz
FIDRES 0.919204 Hz
AQ 1.0879476 sec
RG 101
DW 16.600 usec
DE 6.50 usec
TE 297.7 K
D1 2.00000000 sec
D11 0.03000000 sec
TD0 1
SFO1 125.7753938 MHz
NUC1 13C
P0 3.33 usec
P1 10.00 usec
SI 32768
SF 125.7628175 MHz
WDW EM
SSB 0
LB 1.00 Hz
GB 0
PC 1.40
HN
O
H
N
O
O
HN
O
N
O
OAc
major
7
200
180
160
140
120
100
80
60
40
20
0 ppm

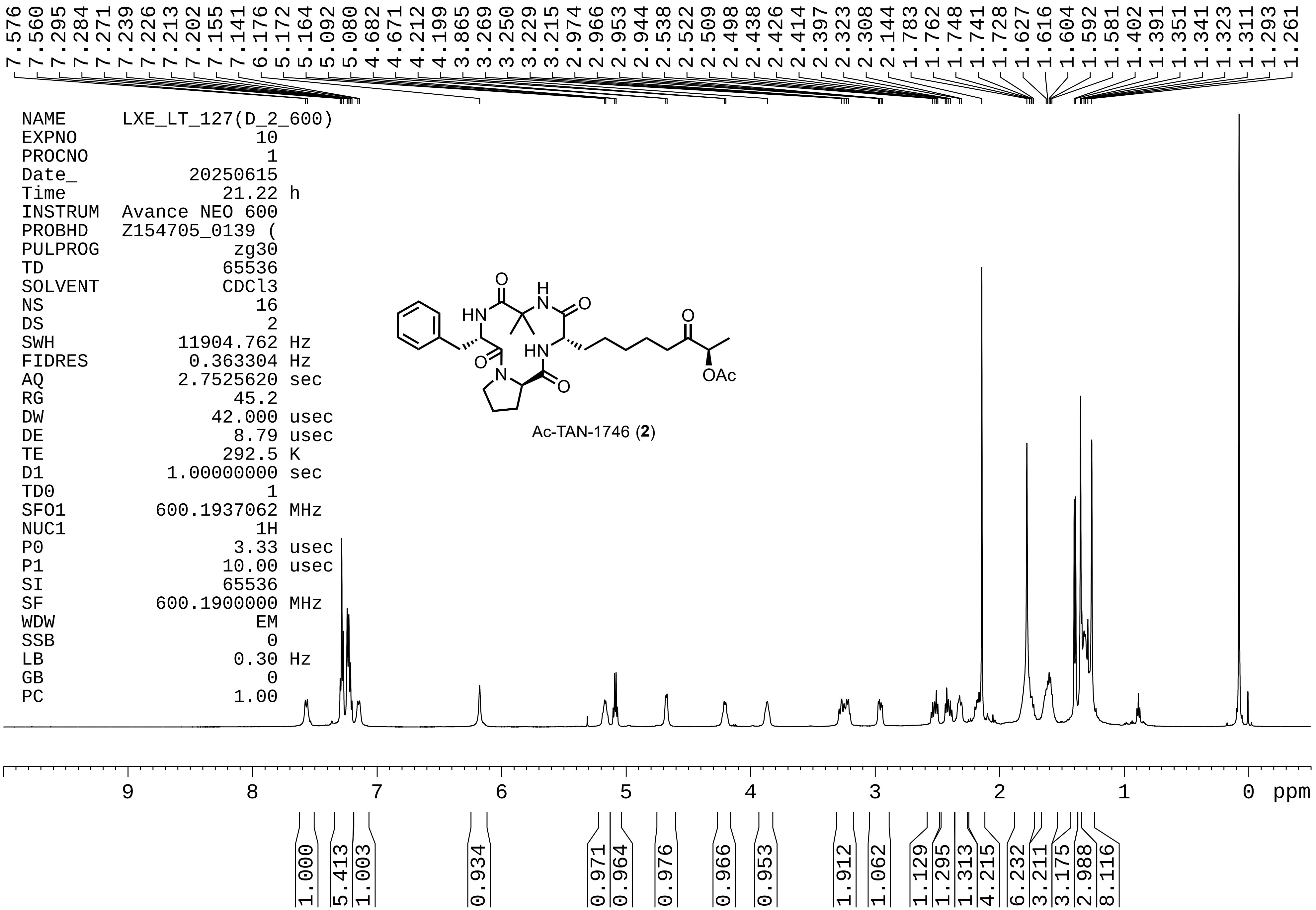
7.576 7.560 7.295 7.284 7.271 7.239 7.226 7.213 7.202 7.155 7.141 6.176 5.172 5.164 5.092 5.080 4.682 4.671 4.212 4.199 3.865 3.269 3.250 3.229 3.215 2.974 2.966 2.953 2.944 2.538 2.522 2.509 2.498 2.438 2.426 2.414 2.397 2.323 2.308 2.144 1.783 1.762 1.748 1.741 1.728 1.627 1.616 1.604 1.592 1.581 1.402 1.391 1.351 1.341 1.323 1.311 1.293 1.261
NAME LXE_LT_127(D_2_600)
EXPNO 10
PROCNO 1
Date_ 20250615
Time 21.22 h
INSTRUM Avance NEO 600
PROBHD Z154705_0139 (
PULPROG zg30
TD 65536
SOLVENT CDCl3
NS 16
DS 2
SWH 11904.762 Hz
FIDRES 0.363304 Hz
AQ 2.7525620 sec
RG 45.2
DW 42.000 usec
DE 8.79 usec
TE 292.5 K
D1 1.00000000 sec
TD0 1
SFO1 600.1937062 MHz
NUC1 1H
P0 3.33 usec
P1 10.00 usec
SI 65536
SF 600.1900000 MHz
WDW EM
SSB 0
LB 0.30 Hz
GB 0
PC 1.00
O
H
HN
N
O
O
HN
O
N
O
OAc
Ac-TAN-1746 (2)
9 8 7 6 5 4 3 2 1 0 ppm
1.000 5.413 1.003 0.934 0.971 0.964 0.976 0.966 0.953 1.912 1.062 1.129 1.295 1.313 4.215 6.232 3.211 3.175 2.988 8.116

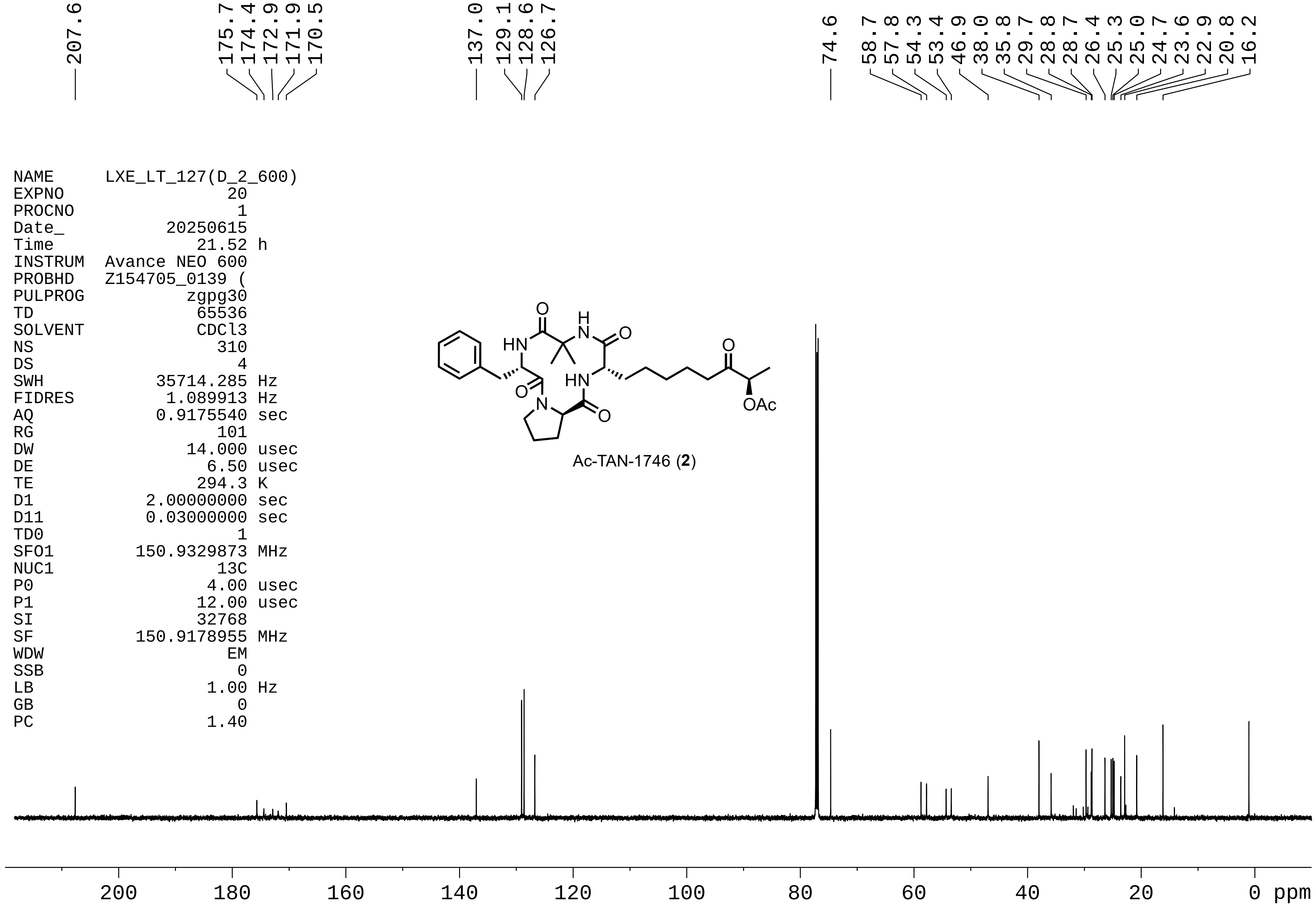

NAME	LXE_LT_127(D_2_600)
EXPNO	20
PROCNO	1
Date_	20250615
Time	21.52 h
INSTRUM	Avance NEO 600
PROBHD	Z154705_0139 (
PULPROG	zgpg30
TD	65536
SOLVENT	CDCl3
NS	310
DS	4
SWH	35714.285 Hz
FIDRES	1.089913 Hz
AQ	0.9175540 sec
RG	101
DW	14.000 usec
DE	6.50 usec
TE	294.3 K
D1	2.00000000 sec
D11	0.03000000 sec
TD0	1
SFO1	150.9329873 MHz
NUC1	13C
P0	4.00 usec
P1	12.00 usec
SI	32768
SF	150.9178955 MHz
WDW	EM
SSB	0
LB	1.00 Hz
GB	0
PC	1.40

Ac-TAN-1746 (**2**)

NAME	TAN-1746	
EXPNO	10	
PROCNO	1	
Date_	20241002	
Time	17.26	h
INSTRUM	Avance NEO 500	
PROBHD	Z119470_0332 (	
PULPROG	zg30	
TD	65536	
SOLVENT	CDCl3	
NS	16	
DS	2	
SWH	10000.000	Hz
FIDRES	0.305176	Hz
AQ	3.2768500	sec
RG	101	
DW	50.000	usec
DE	10.84	usec
TE	296.0	K
D1	1.00000000	sec
TD0	1	
SFO1	500.1530884	MHz
NUC1	1H	
P0	3.24	usec
P1	9.72	usec
SI	65536	
SF	500.1500115	MHz
WDW	EM	
SSB	0	
LB	0.30	Hz
GB	0	
PC	1.00	

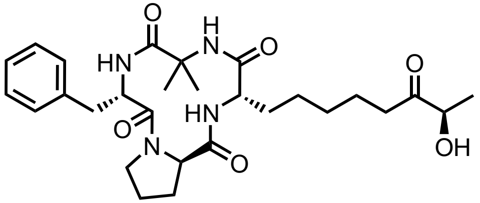

TAN-1746

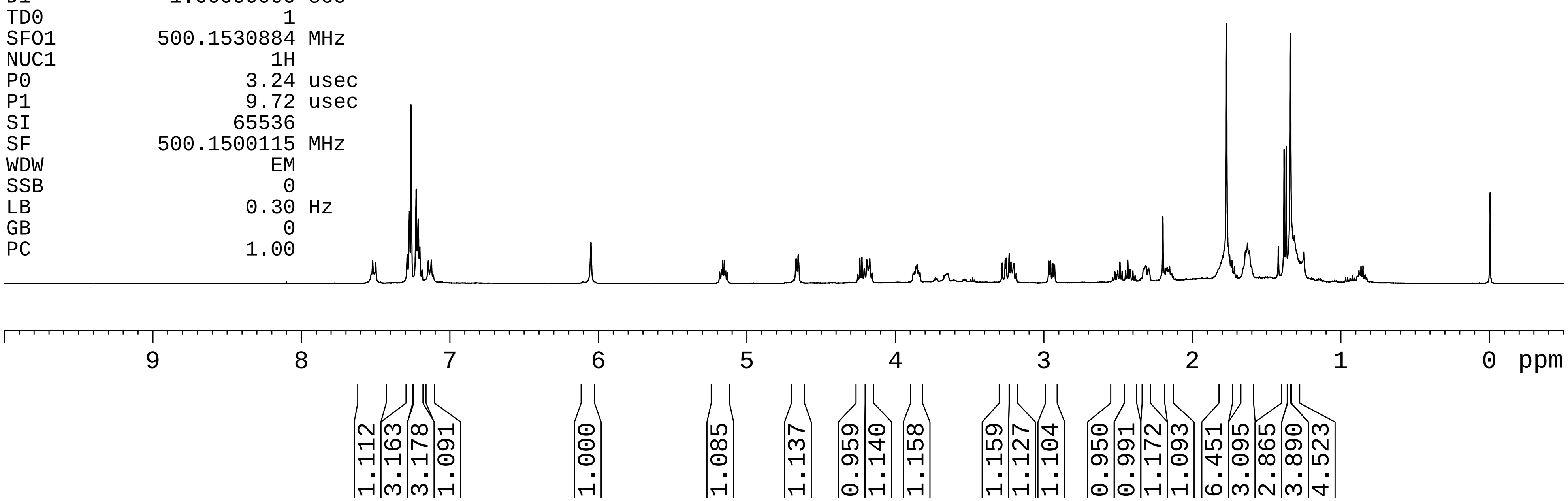

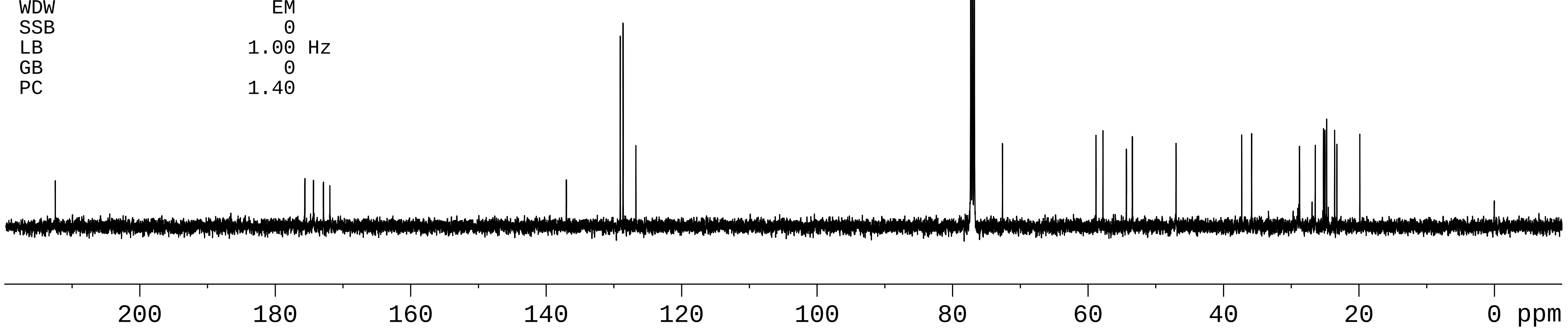

NAME	TAN-1746
EXPNO	11
PROCNO	1
Date_	20241002
Time	17.54 h
INSTRUM	Avance NEO 500
PROBHD	Z119470_0332 (
PULPROG	zgpg30
TD	65536
SOLVENT	CDCl3
NS	520
DS	4
SWH	30120.482 Hz
FIDRES	0.919204 Hz
AQ	1.0879476 sec
RG	101
DW	16.600 usec
DE	6.50 usec
TE	296.5 K
D1	2.00000000 sec
D11	0.03000000 sec
TD0	1
SFO1	125.7753938 MHz
NUC1	13C
P0	3.33 usec
P1	10.00 usec
SI	32768
SF	125.7628175 MHz
WDW	EM
SSB	0
LB	1.00 Hz
GB	0
PC	1.40

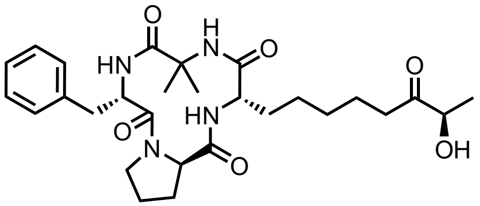

TAN-1746
